# Fluorescent reporters give new insights into antibiotics-induced nonsense and frameshift mistranslation

**DOI:** 10.1038/s41598-024-57597-8

**Published:** 2024-03-22

**Authors:** Mariliis Hinnu, Marta Putrinš, Karin Kogermann, Niilo Kaldalu, Tanel Tenson

**Affiliations:** 1 Institute of Technology, University of Tartu, 50411 Tartu, Estonia; 2 Institute of Pharmacy, University of Tartu, 50411 Tartu, Estonia

**Keywords:** Microbiology techniques, Escherichia coli, Reporter genes, Fluorescent proteins, Translation, Ribosome, Bacterial physiology, Bacterial techniques and applications, Antibiotics, Microbiology

## Abstract

We developed a reporter system based on simultaneous expression of two fluorescent proteins: GFP as a reporter of the capacity of protein synthesis and mutated mScarlet-I as a reporter of translational errors. Because of the unique stop codons or frameshift mutations introduced into the mScarlet-I gene, red fluorescence was produced only after a mistranslation event. These reporters allowed us to estimate mistranslation at a single cell level using either flow cytometry or fluorescence microscopy. We found that laboratory strains of *Escherichia coli* are more prone to mistranslation compared to the clinical isolates. As relevant for uropathogenic *E. coli*, growth in human urine elevated translational frameshifting compared to standard laboratory media, whereas different standard media had a small effect on translational fidelity. Antibiotic-induced mistranslation was studied by using amikacin (aminoglycoside family) and azithromycin (macrolide family). Bactericidal amikacin induced preferably stop-codon readthrough at a moderate level. Bacteriostatic azithromycin on the other hand induced both frameshifting and stop-codon readthrough at much higher level. Single cell analysis revealed that fluorescent reporter-protein signal can be lost due to leakage from a fraction of bacteria in the presence of antibiotics, demonstrating the complexity of the antimicrobial activity.

## Introduction

It is estimated that the rate of different spontaneous translational errors (mistranslation) in bacterial cells is between 10^–5^ and 10^–3^ per codon^1–4^. There are several types and mechanistic causes of translational errors: misincorporation, frame shift and STOP codon readthrough (nonsense suppression). In sum, these result in amino acid substitutions in the protein and/or preterm or late interruption of protein synthesis, which in turn can affect the tertiary structure of the proteins. Most of the errors have a neutral effect. However, mistranslated proteins can lose their function or, in case of membrane proteins, change the cell envelope permeability^5^. At the same time, increased translational accuracy slows down protein synthesis and consequently decreases bacterial growth rate. Thus, an evolved and well tolerated optimum of mistranslation level exists^1^. At certain error-prone sequences, the error frequencies may be significantly higher and, in some cases, mistranslated proteins acquire novel functions that are beneficial for the bacterium^5^. Mistranslation is one mechanism for antibiotic treatment failure and resistance development^6,7^. Mistranslated DNA replication proteins can cause a mutator phenotype, which in turn potentiates antibiotic resistance^5,8–12^. Changes in translational fidelity can also affect the virulence of pathogens^13^. Higher mistranslation rate can cause antigenic variability due to changes in the cell surface proteome, which can help the pathogen to evade recognition by the host’s immune system^14^.

Several ribosome-binding antibiotics are known to cause mistranslation^15,16^. Aminoglycosides are clinically relevant bactericidal antibiotics which interact with the decoding region of the 30S ribosomal subunit. While the exact cause of the bactericidal action of aminoglycosides is not known, it has been explained by increased translational errors, which in turn cause accumulation of protein aggregates that leads to a leaky membrane, further aminoglycoside accumulation inside the cell, and oxidative stress^17–20^. Oxazolidinones and chloramphenicol that bind to the 50S ribosomal subunit cause significant frameshifting and nonsense suppression^16^. Viomycin of the tuberactinomycin family shuts down proofreading-based error correction of protein synthesis and enhances mistranslation^21^.

Four different macrolide antibiotics have been demonstrated to cause stop codon readthrough^15^. Macrolides are bacteriostatic antibiotics that bind to the nascent peptide exit tunnel of the of 50S ribosomal subunit and interact with the growing polypeptide. Ketolide sub-family of this antibiotic class induces ribosomal frameshifting. This happens in the regulatory leader peptide of the macrolide resistance gene *ermC* and triggers its expression^22,23^.

Mistranslation has been studied using immunoblotting, radiolabelled amino acid incorporation, proteomics-based assays etc.^24–30^. Different methods of studying mistranslation have been extensively reviewed elsewhere^31^. Several mistranslation reporters have been developed based on *lacZ*, luciferase or single fluorescent proteins, where stop-codons or frameshift mutations have been introduced into the reporter gene^7,15,16,32–35^. While these are no doubt useful, the lacZ and luciferase-based reporters only allow to investigate the bacterial population in bulk and do not reveal differences between individual bacteria. The production of the reporter protein is influenced by both bacterial metabolic activity and translation-inhibiting antibiotics; however, these factors cannot be taken into account when using single fluorescent protein reporters. Dual-fluorescent reporters have a second reporter protein as an internal control. That allows to consider the overall protein synthesis capacity and metabolic activity of individual cells^36–38^. Dual-fluorescent reporters based on fusion proteins have been used to quantitate stop-codon readthrough and frameshifting^38,39^. Reporter protein synthesis in such a system is highly dependent on the translation efficiency of the first protein, which can be a disadvantage especially when analysing antibiotics. Importantly, bacterial autofluorescence may also significantly change during microbial stress, such as antibiotic treatment^40,41^. Therefore, autofluorescence measurements are necessary to estimate reporter signals correctly. Using red fluorescence as reporter signal is advantageous due to the low autofluorescence background, in contrast to green, blue, and orange fluorescence, spectra of which overlap with various cellular metabolites prone to changing levels^40^. A robust constitutive promoter inducing the reporter system allows to use the reporters in infection and antibiotic treatment models.

Here we have developed a set of dual-fluorescent reporters, to measure stop-codon readthrough and translational frameshifting. We used these reporters in *Escherichia coli* laboratory strains and clinical isolates to find how different environments and stress conditions, e. g. growth in human urine, affect mistranslation levels. We tested them in the presence aminoglycoside antibiotic amikacin that is known to cause mistranslation and applied it to characterize macrolide antibiotic azithromycin (AZI) that has not been studied in this aspect. Single cell analysis revealed us bacterial heterogeneity at near-MIC concentrations of these antibiotics that must be considered while interpreting reporter gene expression.

## Materials and methods

### Plasmid construction and bacterial strains

All in silico plasmid design and confirmation procedures were performed using SnapGene software version 3.2.1 (www.snapgene.com). Plasmids were constructed following the strategy used for design of the reporter for detection of chloramphenicol-induced ribosomal stalling^42^. Low-copy pSC101 vector backbone with ampicillin resistance for selection was used. *GFPmut2* (indicated as GFP throughout this paper) and *mScarlet-I* (iSc) were expressed under two consecutive promoters: a constitutive Ptet promoter and a heat shock-inducible PdnaK1 promoter to increase expression in the presence of antibiotics or other stress factors (Fig. S1). rrnB T2 terminator was inserted in front of the ampicillin promoter to reduce beta-lactamase overproduction due to unspecific transcription resulting in the appearance of microsatellite colonies. T74I mutation was introduced into mScarlet gene resulting in a faster maturing mScarlet-I^43^ (Fig. S2).

**Figure 1:**
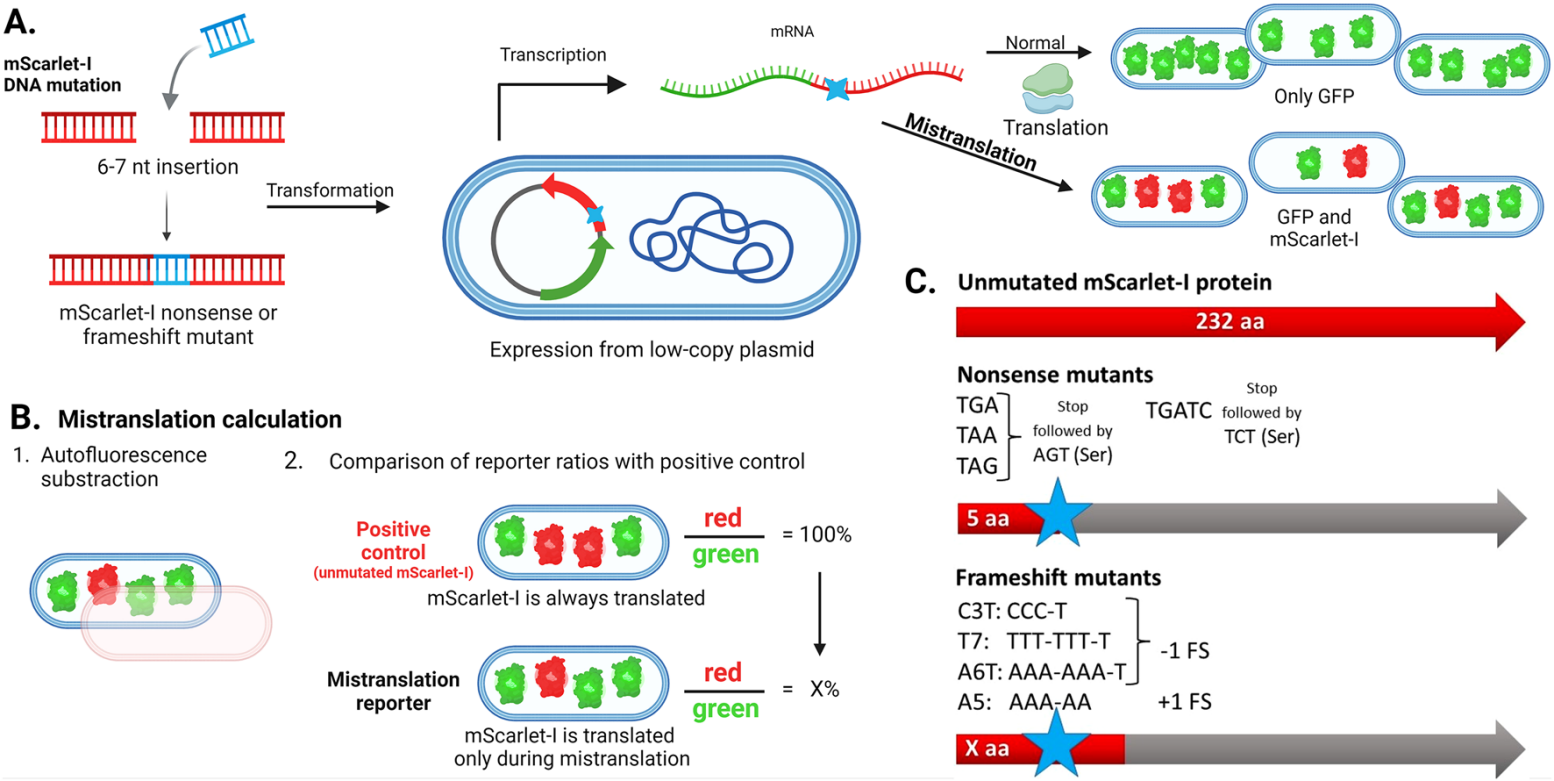
Mistranslation reporter setup. (**A**) Nonsense (stop codon readthrough) and frameshift mutations were inserted into the mScarlet-I gene. Reporter proteins GFP and mScarlet-I are expressed constitutively from a low-copy plasmid carrier on a single mRNA. During accurate translation only GFP is produced. mScarlet-I is only produced during frameshifting (FS) or stop codon readthrough (NS). (**B**) Calculation of mistranslation levels. (**C**) Unmutated mScarlet-I protein serves as the positive control. Insertions were made in the mScarlet-I gene to create a frameshift or stop-codon mutation. + 1/− 1 FS represents the frameshift direction, which can be detected with this reporter system.

Stop-codons or frameshift mutations were introduced into mScarlet-I gene (Fig. 1). Plasmid map is shown in Fig. S3. Unmutated mScarlet-I was used as a positive control and same vector plasmid without mScarlet was used as the negative (autofluorescence) control. Amino acid change or insertion in this region likely does not affect mScarlet-I fluorescence significantly, as the insertion of tryptophan and serine residues in the mutation site did not decrease mScarlet-I fluorescence (Fig. S2). All plasmids were constructed using the circular polymerase extension cloning (CPEC) protocol^44^. FavorPrep™ GEL/PCR Purification Kit (Favorgen Biotech Corp, Taiwan) was used for extracting PCR products from agarose gel. All reporter sequences were verified by sequencing.

CPEC products were transformed into DH5α and selected on ampicillin-containing LB-agar (BD, USA) plates. Plasmids were purified from overnight cultures using commercial plasmid purification kits (ZymoPURE plasmid miniprep kit, Zymo Research, Irvine, USA; FavorPrep™ Plasmid DNA Extraction Mini Kit, Favorgen Biotech Corp, Taiwan). Purified plasmids were transformed into *E. coli* laboratory strains by heat shock [DH5α, MG1655 (wild-type (WT) and error-prone (*ram*) ΔL31AB (ΔL31)^45^), BL21 DE3, MC361 WT with *ram* and error-restrictive (*res*) mutants], and clinical isolates [CFT073 (uropathogen), DSM1103 (standard pathogenic strain for antimicrobial susceptibility testing) and Nissle (probiotic)] by electroporation. Bacterial plasmids and strains are listed in Tables S1 and S2, respectively. 15% glycerol stocks were prepared from overnight cultures of single colonies, flash frozen in liquid nitrogen and stored at -80°C. Dimethyl sulfoxide (DMSO) stocks were prepared as follows: reporter cells were grown aerobically in filter-sterilized BD Difco™ LB Broth, Lennox (LB) (BD, USA) medium at 37°C to OD_600_ ~ 0.5, chilled on ice and mixed with final concentration of 8% DMSO. Stocks were flash frozen in liquid nitrogen and stored at − 80 °C.

### Spontaneous mistranslation in different media

#### Medium preparation

BBL™ Mueller Hinton II Broth (Cation-Adjusted) (MHB) (BD, USA) was boiled for 1 min in a microwave oven, cooled and then filter-sterilized with a 0.2 µm filter prior to experiments.

#### Human urine preparation

Mid-stream human urine was collected from 6 healthy volunteers (3 male, 3 female), sterile filtered (prefiltered 0.45 µm, then 0.2 µm) and stored at 4 °C up to 3 days. Before experiment the urine samples were refiltered in case of sediment appearance, and then pooled in equal volumes. 3 different batches of urine were analysed.

#### Ethics declaration

All methods were carried out in accordance with relevant guidelines and regulations. Ethics approval is deemed unnecessary according to national regulations. Informed consent forms were obtained from the volunteers before urine sample collection. Urine was collected from healthy volunteers. These samples were kept anonymous and were not used for generating any personal data. In accordance with Estonian Personal Data Protection Act (Passed 12.12.2018) no additional approvals for sample collection were needed. We confirm that during collection of human urine no human cells or tissues were isolated and only cell-free samples were used. Pooled urine samples were used as a growth substrate for bacteria in laboratory experiments. The results of these experiments were related to bacterial cells only and no assessment of the individual properties of collected urine was made. This was communicated to the healthy volunteers via the information sheet.

#### Bacterial cultures and analysis

Bacterial cells were streaked from glycerol stocks onto LB-agar plates containing 100 µg/ml of ampicillin (Amp) or carbenicillin (Cb). Single colonies were picked and inoculated into 96-well plates containing 100 µl of medium with 100 µg/ml of Amp using a sterile toothpick. Plates were incubated aerobically using a microtiter plate shaker-incubator at 750 rpm (Cole-Parmer™ Stuart™, UK) or microtiter plate reader Synergy Mx (BioTek Instruments, Inc., USA) at 37 °C for 24 h. 24 h precultures of MC361-derived strains were diluted 100-fold into fresh medium and fluorescence was recorded with microtiter plate reader Synergy Mx over 24 h. In other experiments precultures were diluted to about 10^6^ CFU/ml into fresh medium (100 µl final volume) and incubated in the same conditions for another 18 h. Cultures were diluted into sterile filtered 1X phosphate-buffered saline (PBS) and analyzed immediately with flow cytometry with Attune™ NxT Acoustic Focusing Flow Cytometer (Thermo Fisher Scientific, USA) (green fluorescence: λ_ex_ 488 nm/λ_em_ 530/30 nm; red fluorescence: λ_ex_ 561 nm/λ_em_ 585/16 nm (MHB experiments) or 590/40 (urine experiments)). OD_600_, green fluorescence (λ_ex_ 485/9 nm/λ_em_ 510/9 nm, gain 80) and red fluorescence (λ_ex_ 569/13.5 nm/λ_em_ 600/17 nm, gain 100) were recorded with platereader.

### Aminoglycoside-induced mistranslation

We tested our reporters with aminoglycoside antibiotic amikacin hydrate (AMI; Sigma-Aldrich Chemie GmbH). Stock solution of 25 mg/ml was prepared in Milli-Q water and stored at 4 °C. Conditional minimum inhibitory concentrations (MICs) were determined according to the experimental procedure below (not by standard MIC protocol) and determined visually after incubating on 96-well plates in 100 µl final volumes aerobically at 37 °C for 20 h. Conditional MICs relevant for this study are listed in Table S3. Other aminoglycosides were tested during preliminary experiments, results of which are shown in supplementary data and are referred to in relevant sections in the results.

Filter-sterilized M9 minimal medium (48 mM Na_2_HPO_4_, 22 mM KH_2_PO_4_, 9 mM NaCl, 19mM NH_4_Cl, 0.1 mM CaCl2 and 2 mM MgSO4) with 0.2% glucose^46^ was used as the growth medium. Precultures were started from DMSO stocks and grown aerobically at 37 °C in 3 ml of growth medium containing 100 µg/ml Amp to OD_600_ ~ 0.5 (exponential preculture). Cultures were diluted in the same medium to OD_600_ of 0.1. 50 µl of culture OD_600_0.1 was added to 50 µl of fresh medium (without Amp) with aminoglycoside, resulting in final concentrations of 8…0.5 µg/ml (2…1/8 conditional MICs) and a starting OD of 0.05. Plates were incubated aerobically at 37 °C using a microtiter plate shaker-incubator (Cole-Parmer™ Stuart™, UK) or microtiter plate reader Synergy Mx (BioTek Instruments, Inc., USA). Samples (1–1.5 µl in each timepoint) were taken for flow cytometry every hour 1…6 h and at 18 h, diluted into sterile filtered 1 X PBS and analysed immediately.

For microscopy, precultures were prepared as described above. Exponential preculture was diluted to OD_600_ ~ 0.05 in 3 ml of M9 medium containing 2 µg/ml (½ MIC) AMI and without antibiotics. Tubes were incubated aerobically at 37 °C for 4 h. Antibiotic-treated sample was concentrated 20X before imaging on 1% agarose (1:1 mix of low-melt and high-melt agarose) in 1 X PBS pads. Microscopy images were taken using Zeiss Axio Observer Z1 microscope with 100 × oil objective. Phase contrast, green fluorescence (λ_ex_ 474/28 nm/λ_em_ 527/54 nm, 18% light source intensity, 70 ms exposure time) and red fluorescence (λ_ex_ 572.5/25 nm/λ_em_ 645/60 nm, 18% light source intensity, 6.2 s exposure time) were recorded. Fluorescence intensities were adjusted during post-processing for enhanced visibility. Display properties of all samples were manipulated equally.

### Azithromycin-induced mistranslation

25 mg/ml stock solution of azithromycin (AZI) (Carbosynth, UK) was prepared in 96% ethanol and stored at + 4 °C. (Cation-adjusted) MHB medium was prepared as described above. CFT073 with mistranslation plasmid from a single colony was added to 100 µl of medium containing 100 µg/ml Amp with a sterile toothpick and incubated aerobically overnight at 37 °C using a microtiter plate shaker-incubator (Cole-Parmer™ Stuart™, UK) at 750 rpm. Exponential preculture was prepared as follows: 100 × dilution from the overnight culture was made into fresh medium without Amp and incubated in the same conditions as previously for 3 h. Exponential preculture was diluted 10 × into medium without Amp with 32…0.5 µg/ml final concentration of AZI and without antibiotics. AZI was applied at concentrations between 4 and 1/16 of MIC (Table S3). Microtiter plates with AZI were incubated as previously for 4 h, and then chilled on ice. 20% final concentration of glycerol was added to each well and plates were frozen at -80 °C until analysis with Attune™ NxT Acoustic Focusing Flow Cytometer (Thermo Fisher Scientific, USA) (green fluorescence: λ_ex_ 488 nm/λ_em_ 530/30 nm; red fluorescence: λ_ex_ 561 nm/λ_em_ 585/16 nm.

### Calculating mistranslation rate

Red and green fluorescence signals were recorded for each reporter using platereader, microscopy, or flow cytometry. Flow cytometry data were analyzed using FlowJo™ Software version 10.7 (BD Life Sciences, USA). Green fluorescence and side scatter plots were used for gating cell populations—gating strategy is shown on Fig. S4A. No compensation for fluorescence spillover was used (Fig. S4B). Gating bacterial cells according to side and forward scatter only is not possible with our equipment (Fig. S4C). Geometric means of red and green fluorescence were used in analyses. Ratios of red:green fluorescence (R:G) were calculated for each reporter. R:G of the pSC101-GFPmut2-mScarlet-I (GFP + iSc +) containing bacteria (Table S1; unmutated mScarlet-I gene) was equalized to 100%. R:G values of the samples bearing mutant mScarlet-I were expressed as percentages of the R:G of the GFP + iSc + bearing cells. Red autofluorescence of bacteria expressing no mScarlet-I and containing either plasmid pSC101-GFPmut2-ΔmScarlet or pSC101-AmpR EV was measured in each experimental condition and *E. coli* strain. It was subtracted from the red fluorescence of the reporter-containing bacteria before the R:G calculations. Mistranslation calculation is shown schematically in Fig. 1B. Data are based on the average of at least 3 experiments. For statistical analyses we carried out unpaired t test with Welch correction assuming individual variance for each group (α = 0.05). No multiple comparison corrections were made. Calculated P values have been reported in Tables S4–S7.

### Software for creating figures

Drawn images were created with BioRender (www.biorender.com). Graphs were constructed and statistical analyses performed with GraphPad Prism version 10.0.3 (GraphPad Software, USA, www.graphpad.com). Microscopy images were visualized with ZEN 3.8 (Carl Seizz Microscopy GmbH, Germany). Flow cytometry data graphs were created with FlowJo™ Software version 10.7 (BD Life Sciences, USA, www.flowjo.com). Final figures were constructed with Affinity Publisher 2.2.1 (Serif (Europe) Ltd., UK, affinity.serif.com).

## Results

### Mistranslation level depends on strain, specific reporter, and time of measurement

We developed a set of dual-fluorescent mistranslation reporters for *E. coli* (Fig. 1). Mutated red fluorescent protein mScarlet-I (iSc) gene^43^ was used as the reporter of mistranslation and co-transcribed with Green fluorescent protein (GFPmut2) gene serving as an indicator for bacterial capacity of protein synthesis. A similar plasmid bearing unmutated mScarlet-I and GFPmut2 genes was used as a comparison in all experimental conditions to measure the level of red fluorescence that would be produced by productive translation. As autofluorescence levels can change depending on conditions (Fig. S5) red autofluorescence of bacteria expressing no iSc was measured separately and subtracted from the red fluorescence signal of reporters. Ratio of the adjusted red and green fluorescence signals (R:G) in the cells bearing intact iSc was equalized to 100%. Mistranslation was estimated as the percentage of red:green fluorescence ratio in cells having mutant mScarlet-I reporter from the red:green ratio of bacteria containing unmutated iSc. (Fig. 1B).

Stop codon (nonsense, NS) and frameshift (FS) mutations were introduced into the 6^th^ codon of the iSc gene (Fig. 1C). Amino acid insertion in this region did not decrease iSc fluorescence (Fig. S2). Serine codon AGT was inserted after all stop codons, except for the nonsense reporter TGATC, which is followed by serine codon TCT in order to test how the nucleotide following the stop codon affects NS misreading. FS mutation results in a stop codon appearance downstream of the mutation site. Full-length iSc is produced only when stop codon is ignored or the correct reading frame is restored during translation. Frameshifting at slippery sequences can occur in both directions. However, our system reports only direction indicated in Fig. 1C, as the reverse FS would result in a random 10–25 amino acid polypeptide unlikely to produce any red fluorescence. Abbreviations for each reporter are explained in Fig. 1C and Table S1.

**Figure 2:**
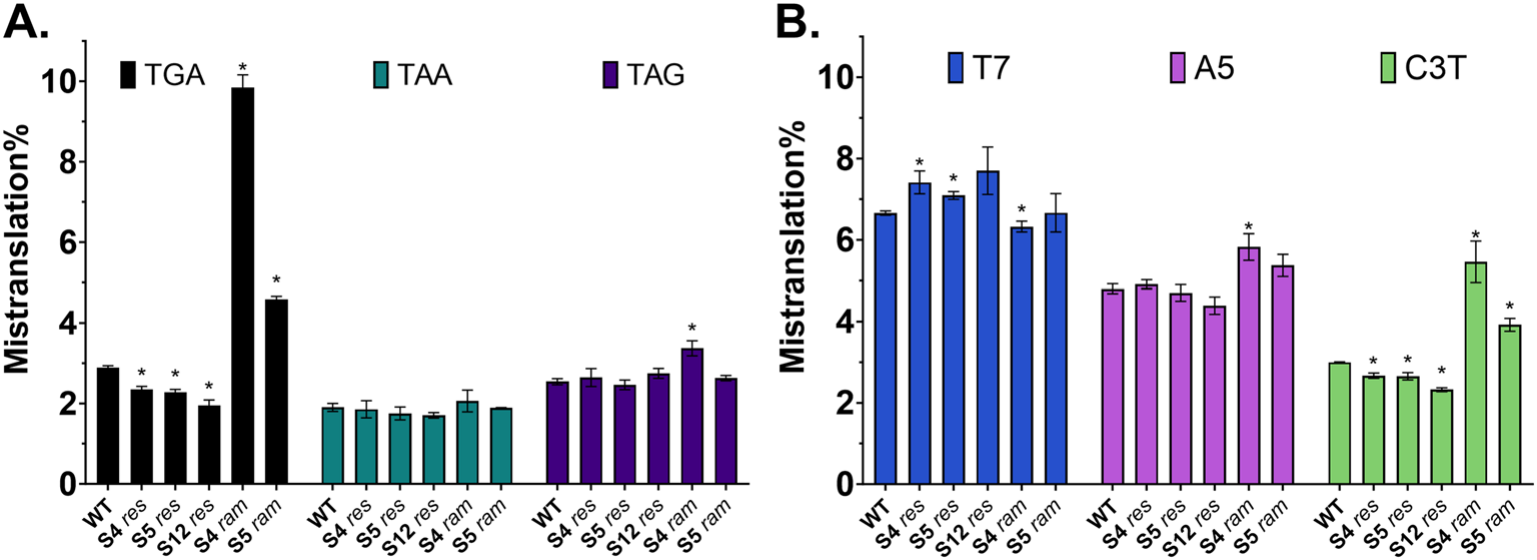
Mistranslation levels of error-restrictive (res) and error-prone (ram) E. coli strains after 24 h of incubation. (**A**) Stop-codon readthrough of TGA-stop is most affected by the ribosomal mutations tested. (**B**) Frameshifting levels of A5 and C3T reporters are increased in ram strains, surprisingly however, T7 frameshift is slightly elevated in res strains. Platereader data. Mean ± SD (3 biological replicates) is shown. *P value of the difference to WT strain < 0.05.

In order to verify that the reporter can be used to detect mistranslation changes we carried out experiments with different *E. coli* error-prone and error-restrictive ribosomal mutant strains^45,47,48^. A set of *ram* and *res* mutants revealed in accordance with previously published data^47,48^ that TGA readthrough is significantly higher than WT in *ram* strains and lower in *res* strains (Fig. 2A, Table S4). Surprisingly, TAG readthrough was only slightly increased in *ram* strains, whereas *res* strains showed no changes compared to WT. There were no significant effects in case of TAA stop for neither *ram* nor *res* strains*.* In case of frameshift reporters (Fig. 2B, Table S4) C3T showed significantly higher levels in *ram* strains and slightly lower levels in *res* strains. In reporter A5 *ram* strains had only slightly elevated frameshift levels. Conversely, *res* strains showed marginally elevated T7 frameshift levels compared to WT, while *ram* strains maintained similar levels to WT. T7 signal in *res* mutants seems to increase in stationary phase, whereas other reporters maintain their relative levels throughout the time course (Fig. S6).

Altogether, these results indicate that mutations in the ribosomal proteins S4, S5, and S12 cause specific changes in translational fidelity, not a global shift. The reporters also demonstrated that different mistranslation events happen at different frequencies, the induction rates are different for each individual reporter (Fig. 2 and Fig. S7) and levels remained similar in different media (Fig. S7). The highest differences in mistranslation level between the WT and ΔL31 strain were detected during the first few hours of growth (Fig. S8).

**Figure 3:**
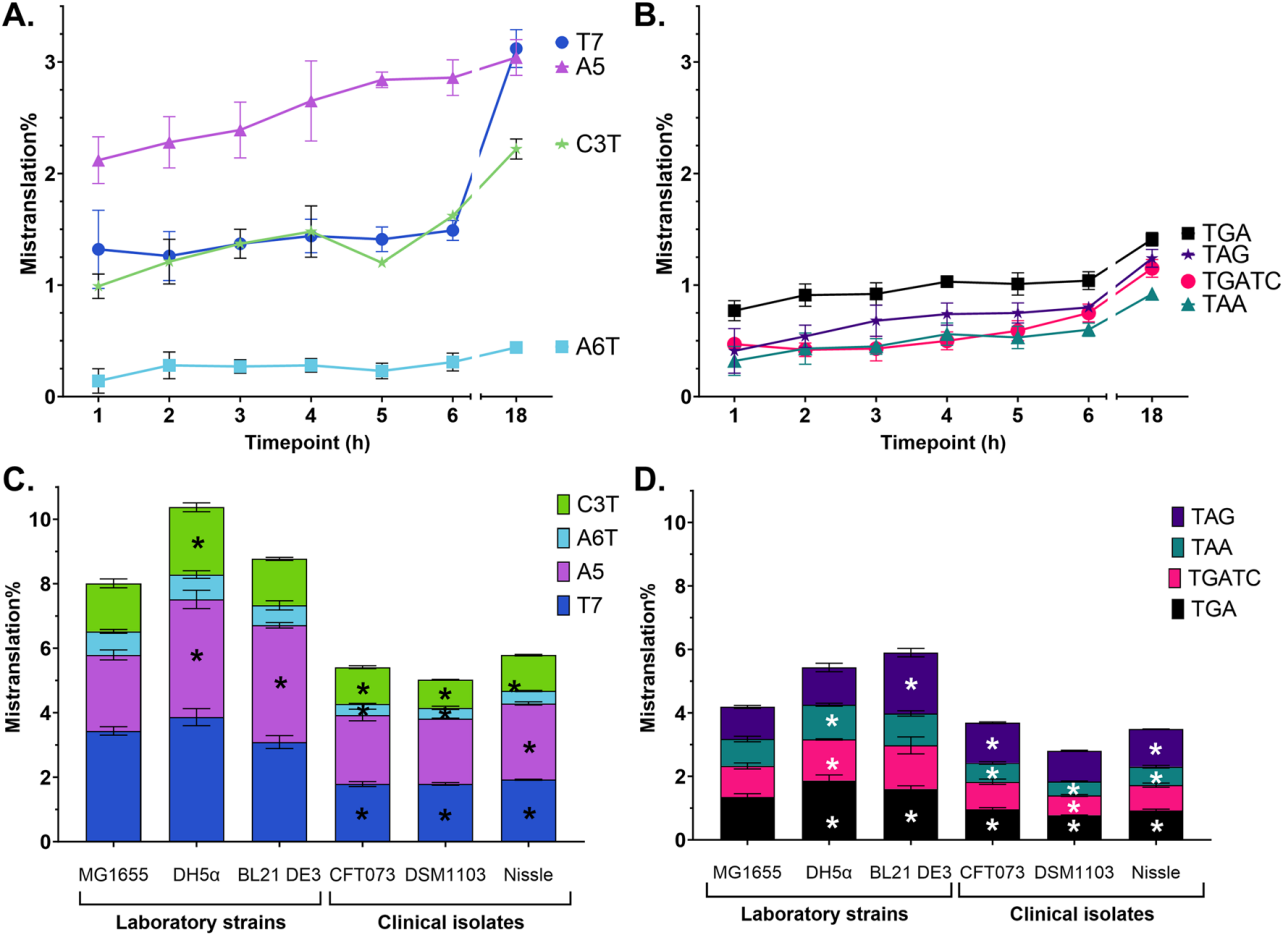
Mistranslation rates depend on specific sequence, growth time and the strain used. Growth time in M9 minimal medium with 0.2% glucose affects frameshift (**A**) and stop codon readthrough (**B**). Laboratory E. coli strains have both higher frameshift rates (**C**) and stop codon readthrough rates (**D**) compared to clinical isolates in MHB medium after 18 h. Flow cytometry analysis, N = 3, mean ± SD. *P value of the difference to MG1655 strain < 0.05.

Further, we tested mistranslation time-course using different reporters in laboratory *E. coli* strain MG1655 during growth in M9 minimal medium supplemented with 0.2% glucose (Fig. 3A,B). Misreading rates of individual reporters varied up to 10 times. During first hours of growth mistranslation levels remained relatively unchanged, however there was a slight increase during late stationary phase for all reporters. Frameshifting rate of individual reporters was between 0.3 and 4% (Fig. 3A). Stop-codon readthrough levels are approximately 1% (Fig. 3B). TGA stop is the most commonly misread stop codon. TGA followed by an adenosine nucleotide has slightly higher mistranslation rate than TGA followed by thymidine.

Next, we compared different *E. coli* strains in MHB medium at 18 h timepoint. We found that *E. coli* laboratory strains have higher mistranslation rates compared to the clinical isolates tested (Fig. 3C,D). However, the misreading patterns of different reporters generally remain similar between tested strains: highly misread sequences have higher rates in all strains, and rarely misread sequences remain low in all strains. Reporters T7 and A5 have similar frameshift rates around 2% and 4% in clinical isolates and laboratory strains, respectively, and have the highest error rates. Interestingly, BL21 DE3, a strain commonly used for protein production, has approximately 2 times higher TAG readthrough rate than other tested strains. TAA stop has the lowest mistranslation rate in all strains tested. Growth rates do not differ between the strains, except for the laboratory strain DH5α, which has slightly slower growth rate (Fig. S9). *Salmonella enterica* serovar Typhimurium SME51 produced similar mistranslation levels to *E. coli* strain CFT073 (Fig. S10).

### The type of standard growth medium has minimal effect on mistranslation rates whereas human urine induces translational frameshifting

**Figure 4:**
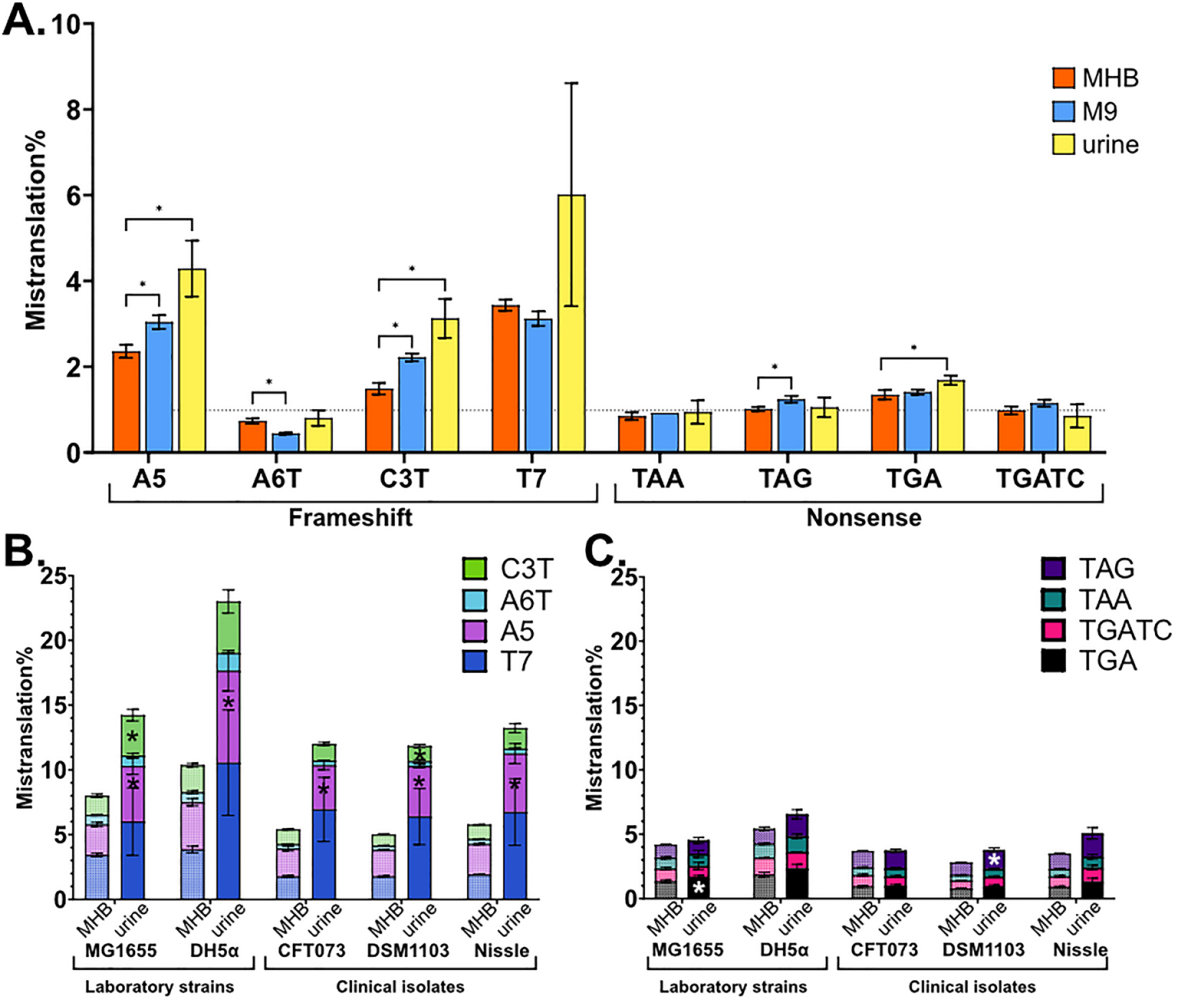
Mistranslation rates of E. coli after 18 h of incubation. (**A**) Laboratory strain MG1655 in 3 different growth media. Flow cytometry analysis, N = 3, mean ± SD. Frameshift (**B**) and stop codon readthrough (**C**) levels in E. coli clinical isolates and laboratory strains in human urine compared to MHB (patterned columns). Flow cytometry data. Means of 3 different batches of urine were compared. *P value of difference to MHB medium < 0.05.

Next, we tested if growth media could affect mistranslation levels. While different media produce different growth rates and overall protein production levels, the mistranslation differences detected in *E. coli* MG1655 in M9 and MHB media were negligible (Fig. 4A and Fig. S7). At the same time, results using frameshift reporters A5, C3T and T7 suggested that growth in human urine, an environment encountered by uropathogenic organisms, most common of which is *E. coli*^49^, can induce mistranslation, which encouraged us to further investigate this phenomenon.

We measured mistranslation levels in different *E. coli* strains upon growth in human urine. We found that frameshifting rates increased in all tested strains in pooled human urine (Fig. 4B,C, Table S5). Average frameshifting rates in urine increased up to 4 times compared to MHB, however induction varied between reporters and strains. Uropathogenic strain CFT073 demonstrated increased frameshift of reporters A5 and T7. Average stop codon readthrough increased only slightly and was up to 1.5 times higher in urine. It is important to note that the variability between results of individual experiments conducted in urine was much higher than in MHB. This can be explained by higher variability between different batches of pooled human urine and unknown factors that can affect mistranslation in it when compared to identical batches of standard MHB medium. Due to high variability, the observed increases of the average levels of mistranslation in urine were in many cases statistically insignificant, e.g. the T7 frameshift that demonstrated the largest increase of average misreading (Fig. 4, Table S5). Of note, laboratory *E. coli* strains had poorer growth in urine compared to the clinical isolates.

### Amikacin increases stop-codon readthrough

**Figure 5:**
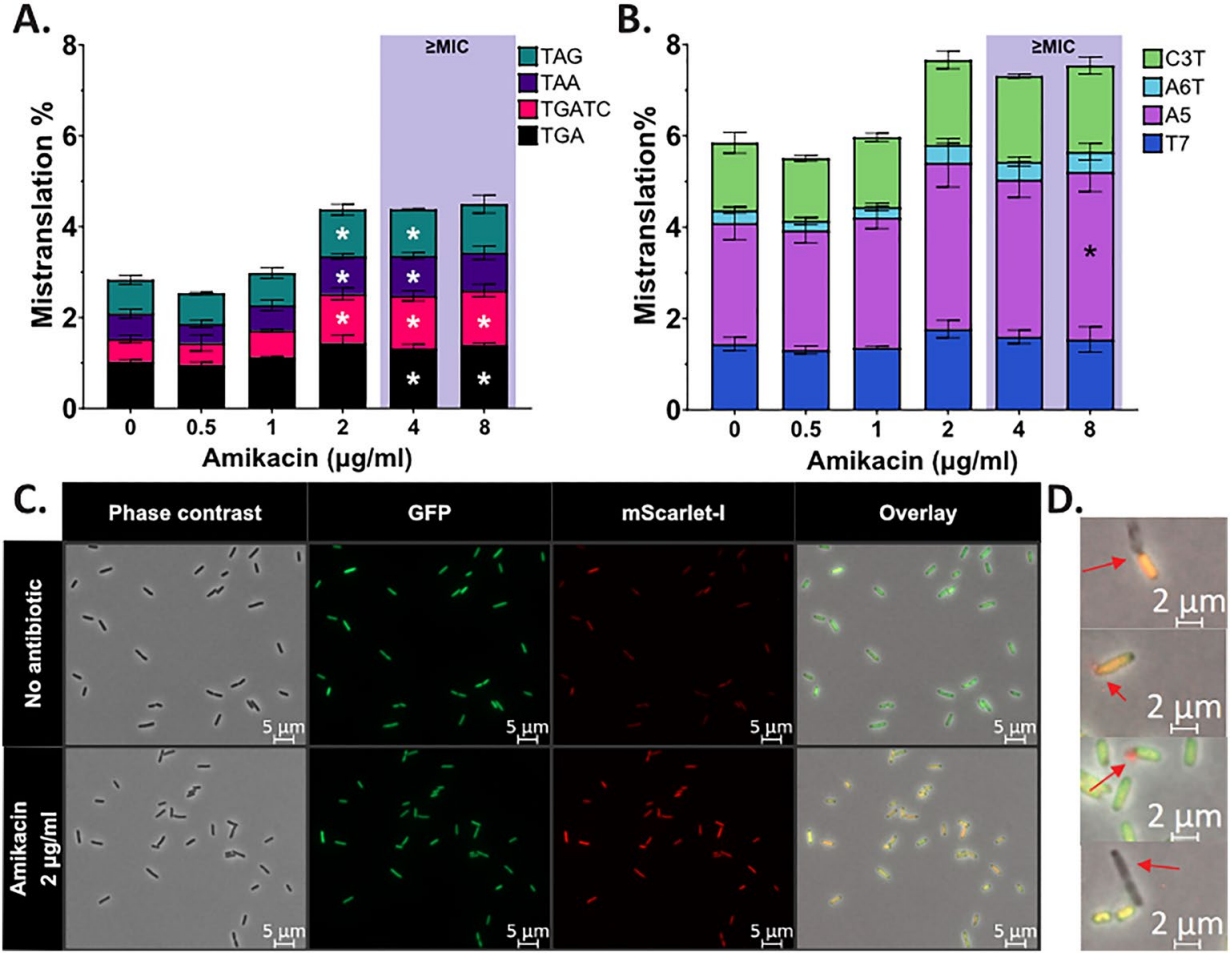
Mistranslation levels increase during aminoglycoside amikacin treatment. (**A**) Stop codon readthrough increases with amikacin in a concentration-dependent manner. (**B**) Frameshift also increases with amikacin, however to a lesser extent. (**C**) Microscopy analysis of TGA reporter cells confirms increased mistranslation with amikacin. (**D**) A few bacterial cells in antibiotic-treated samples have a fluorescent halo near a pole (indicated by red arrows). AMI-treated samples also have a higher number of non-fluorescent cells (top and bottom images). This indicates very probably leakage of the cell content, including fluorescent proteins. MG1655 cells were treated with amikacin for 4 h in M9 minimal medium with glucose before analysis with flow cytometry, N = 3, mean ± SD (**A**,**B**) or fluorescence microscopy (**C**,**D**). Amikacin MIC in these conditions was 4 µg/ml. *P values of differences to no-treatment control < 0.05.

Next, we decided to study how aminoglycoside antibiotics, which are known to have a bactericidal effect by increasing mistranslation, affect mistranslation reporter signals. Our mistranslation reporters confirm that aminoglycosides increase stop codon readthrough, which increased about 2 times overall at the concentrations starting from 1/2MIC (Fig. 5A and Fig. S11, Table S6). Frameshifting is induced to a lesser extent (Fig. 5B and Fig. S11, Table S6). Mistranslation reporter signal correlates with AMI concentration within the first few hours of antibiotic treatment (Fig. S12). The highest overall increases in mistranslation can be seen with reporters, which have the lowest basal levels, e.g. TAA stop nonsense reporter. Increased mistranslation in response to amikacin was also detected with fluorescence microscopy (Fig. 5C, Fig. S13).

Of note, during flow cytometry analysis we witnessed about 30% decrease of GFP signal and about 10% decrease of iSc signal at the highest AMI concentrations in case of the positive control (GFP + iSc +), whereas red fluorescence of reporters slightly increased (Fig. S14). We also spotted some bacteria with the signs of cell content leakage in AMI-treated samples (Figs. 5D, Figs. S13A, S14). This may indicate that, as soon as antibiotic levels increase, some bacteria are subject to cell death, lose membrane integrity and burst. The cell content, including the fluorescent proteins, leaks out, the bacteria become non-fluorescent and are not detected by flow cytometry. Antibiotic-treated samples also have a higher occurrence of dark spots on the poles of bacteria visible in phase contrast, which might indicate the presence of protein aggregates. As demonstrated by the fluorescence microscopy analysis, red fluorescence increased as a result of the AMI treatment even for the positive control GFP + iSc +, whereas GFP levels remained unchanged (Fig. S13), proving the need to compare ratio of two fluorescence signals to estimate mistranslation correctly. No change in red autofluorescence was detected with microscopy.

### Azithromycin induces mistranslation and population heterogeneity

**Figure 6:**
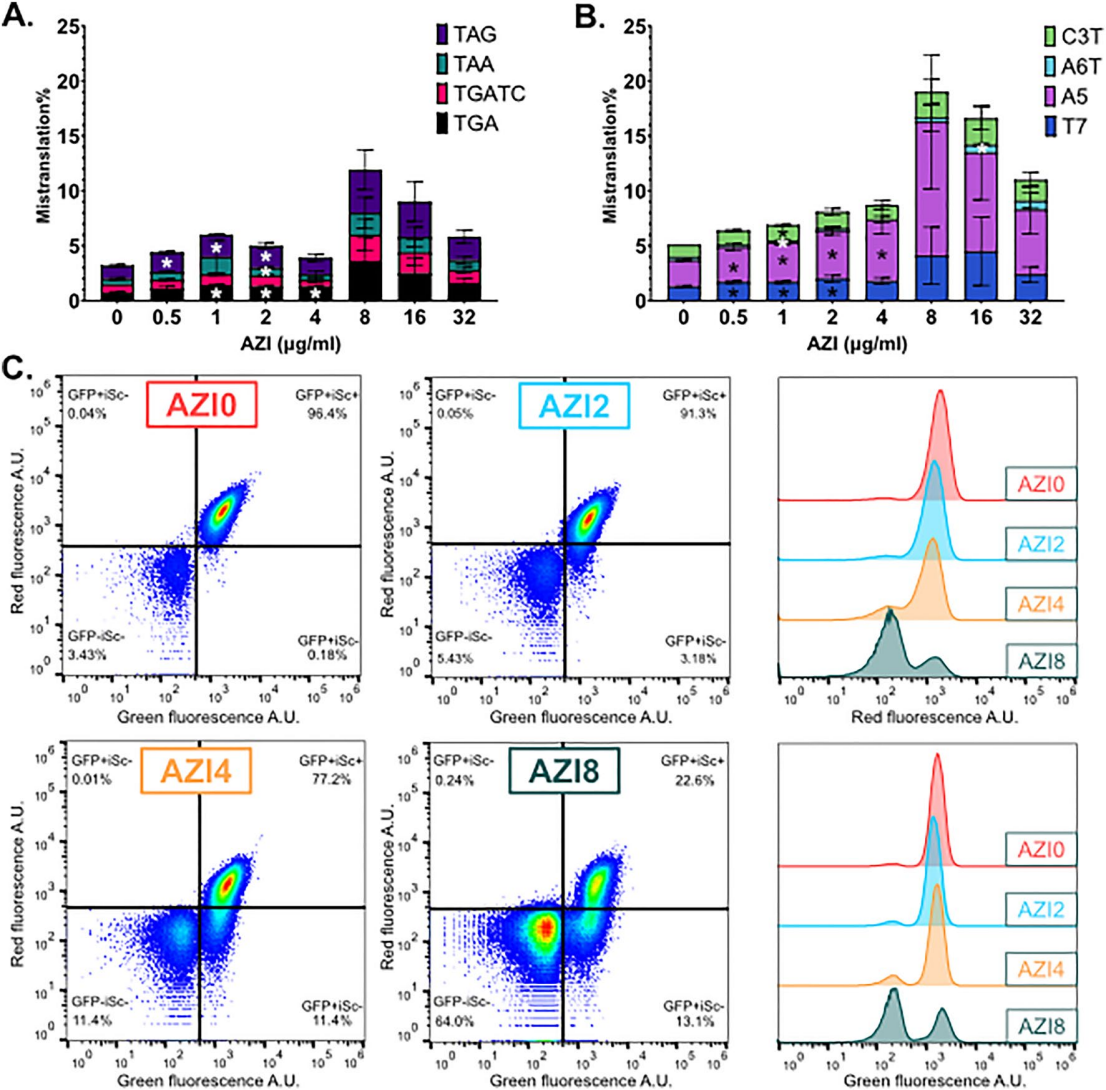
Azithromycin (AZI) induces mistranslation and population heterogeneity in uropathogenic E. coli CFT073. Stop codon readthrough (**A**) and ribosomal frameshifting (**B**) in the presence of azithromycin for 4 h is calculated based on GFP and mScarlet-I ratios of cells carrying respective reporter plasmids and compared to the cells with control plasmids. *P values of differences to no-treatment control < 0.05. (**C**) Flow cytometry analysis of cells with positive control plasmid (GFP + iSc +) expressing GFP and unmutated mScarlet-I reveal heterogeneity of fluorescence signal in AZI-treated samples. Cells were grown for 4 h in the absence (AZI0) or presence of AZI at concentrations 2 µg/ml (AZI2), 4 µg/ml (AZI4) or 8 µg/ml (AZI8) in MHB medium. GFP + iSc-population and non-fluorescent (negative for both signals) populations increase with the antibiotic exposure. Non-fluorescent population overlaps with flow cytometry noise. Histograms show the same data as on dot-plots to compare separately GFP and mScarlet-I signals in different samples. For experiments on panels (**A**) and (**B**) mean and standard deviation of three independent experiments is shown. For panel (**C**) one representative from three independent experiments is shown.

Next, we wanted to investigate how bacteriostatic antibiotic azithromycin affects mistranslation. We determined mistranslation rates of uropathogenic *E. coli* strain CFT073 in the presence of azithromycin. Our mistranslation reporters show that azithromycin induces average stop codon readthrough up to 3 times and ribosomal frameshifting about 4 times (Fig. 6 and Fig. S16). This is far exceeding the mistranslation induction levels seen with aminoglycosides in this study. Highest average induction occurred at concentrations above the MIC, which is 8 µg/ml when measured according to the standard protocol^50^ (Table S3). Conditional MIC determination was not possible due to high starting inoculum in this assay. Maximum mistranslation levels are achieved at 2 × MIC, whereas at higher concentrations the mistranslation levels start decreasing again. As we already observed during testing the effect of urine, the variability of mistranslation between individual AZI-treated samples was high, specially at the AZI concentrations exceeding the MIC. Due to the high variability, the observed increases of mistranslation were in many cases statistically insignificant (Fig. 6A,B, Table S7).

Interestingly, there is an additional peak of stop codon readthrough at 1 µg/ml of AZI, where stop codon readthrough increases about twofold. Induction factors were similar for all the stop codons in the presence of all the AZI concentrations tested. Frameshift rates vary between the reporters and increase the most in A-track and T-track reporters A5 and T7, which also have the highest spontaneous frameshift values. AZI effects on green and red fluorescence signals are shown in Fig. S17.

Of note, mistranslation was calculated based on the average fluorescence of the gated GFP-positive single cell population, as in previous experiments. We noticed already in experiments with AMI some non-fluorescent cells at high antibiotic concentration (Fig. S14). Therefore, we decided to take a closer look to the whole population of bacterial cells carrying GFP and mScarlet-I positive control plasmid. As shown in Fig. 6C, increasing AZI concentration induced population heterogeneity. At 2 µg/ml of AZI, bacteria with reduced or absent mScarlet-I fluorescence appear. Unlike the AMI-treated bacteria (Fig. S15), many of the AZI-treated cells retain GFP and lose only mScarlet-I signal. At concentrations ≥ 4 µg/ml of AZI, a mScarlet-I and GFP double negative sub-population started to increase. These bacteria are overlapping with electronic noise of the flow cytometer on the plot and, therefore, cannot be precisely counted (Fig. 6C). At the same time, increasing concentrations of AMI only slightly increased this population, indicating minor increase of non-fluorescent bacteria (Fig. S15). While it is hard to pinpoint the exact reason for appearance of heterogeneity among AZI-exposed bacteria, it is most likely caused by loss of membrane integrity and leakage of the cell content. While the AMI-treated dark cells had lost both fluorescent marker proteins, the AZI-treated bacteria preferentially leaked mScarlet-I. Thus, we observed disproportionate loss of one of the two reporter proteins in a subset of bacteria in response to AZI and appearance of a dim subpopulation in response to both AZI and AMI. The dark subpopulation has lower side scatter values, indicating a smaller size, but cannot be completely separated from fluorescent cells according to forward or side scatter (Fig. S4C).

We tested whether the reporters could be used in cell culture infection and AZI treatment. We used CFT073 with frameshift reporter T7 for infecting J774 murine macrophages. We found that both GFP and iSc are detectable in infection experiment (Fig. S18), indicating that the reporters are suitable for measuring spontaneous mistranslation during infection. We found high red fluorescence signals inside macrophages at AZI concentration 30 µg/ml, however the signal did not overlap with green fluorescence. Visibly green bacterial cells in those conditions were probably outside of the macrophages and did not show signs of high red fluorescence. This and the previous knowledge of heterogeneity induced by AZI from flow cytometry data highlights the complexity of bacterial response to antibiotic treatment during infection, as well as the limitations of using fluorescence-based reporters in such experiments.

## Discussion

We have developed a dual-fluorescent reporter set that allows to measure frameshift and nonsense mistranslation in *E. coli*. The advantage of our reporter system is the usage of low-background mScarlet-I as the reporter together with GFP that serves as an indicator for capacity of protein synthesis. Fluorescent reporters allow to follow individual variability of bacterial cells. Importantly, we measured and subtracted autofluorescence, which makes estimations of mistranslation more precise, especially during stress-inducing conditions, where autofluorescence levels can increase (Fig. S5)^40,41^.

Our reporter set detected highest mistranslation levels in the presence of macrolide antibiotic azithromycin, which induces both stop codon readthrough and frameshifting 3–fourfold (Fig. 6). Aminoglycosides, which are thought to cause cell death through increased mistranslation, moderately induce mostly stop codon readthrough (Fig. 5, Fig. S11), but the maximum levels remain much lower than those detected with azithromycin. Indeed, macrolides of the ketolide subfamily are known to induce frameshifting that increases expression of the *ermC* resistance gene^23,51^ and very high stop-codon readthrough levels have been detected for macrolides other than azithromycin^15^. Earlier studies have also reported low aminoglycoside-induced stop-codon readthrough and frameshifting^15^, which we confirmed with our reporters. Instead, aminoglycosides induce amino acid misincorporation^16^, which cannot be detected with the reporters included in our set. Azithromycin is bacteriostatic, indicating that certain level of mistranslation is tolerated and is not causing cell death. It has been recently demonstrated that aminoglycosides induce not only single translation errors, but also clusters of errors of up to four amino acid misincorporations in vivo. These error clusters may be the main source of proteotoxic stress, which is involved in the autocatalytic uptake of aminoglycosides^52^. Therefore, aminoglycosides might have a more direct effect on the integrity of bacterial membrane^53^. This in turn causes an increase of antibiotic influx, which potentiates membrane damage, leakage of essential cell contents, and through ribosome binding complete translational arrest, most importantly by blocking initiation^54,55^. Macrolides on the other hand probably do not damage the membrane in such a manner and just obstruct the nascent peptide exit tunnel near the peptidyl-transferase site^23^, causing growth arrest by changing the kinetics of translational processes and increasing mistranslation.

Single cell analysis of bacteria treated with azithromycin revealed that several accompanying or downstream events complicate detection of mistranslation. First, it is possible that mScarlet-I is more sensitive to misfolding than GFP, which might be caused by the slower maturation of the mScarlet-I^56^. Misfolded or otherwise abnormal proteins get preferentially degraded^57^. Secondly, it is possible that antibiotics at a certain concentration and depending on overall physiological state of bacteria induce the generation of reactive oxygen species (ROS)^53^, which can affect fluorescence. Thirdly, if antibiotic action damages membrane integrity, fluorescent proteins can leak out of the bacterial cell^58^. It is also possible that the heterogeneity results from a combination of these events. Overall, we expect that the cells with lost fluorescence are more susceptible to antibiotic action or even dead. Disproportionate loss of one of the two reporter proteins in a subset of bacteria, regardless of whether this happens to the mutated reporters or intact mScarlet-I, has a clear impact on the results. Appearance of a dim subpopulation in a drug-concentration dependent manner limits the utility of the reporter systems and complicates interpretation of the results.

Our results demonstrate that a bacteriostatic drug like azithromycin induces leakage of fluorescent proteins, that probably indicates lethal damage to a part of the population. This observation is in line with previous studies showing that bacteria with low numbers of ribosomes can be killed by macrolides^59,60^. At the same time, fast-growing cells with abundant ribosomes can reach a state where efflux and other stress response mechanisms are induced to keep intracellular azithromycin concentration low and repair the damage caused by the antibiotic^60^. In addition, it has been shown for some macrolide family antibiotics, that the prolonged translation interruption caused by the slow rate of antibiotic disassociation is behind killing of bacteria, while fast dissociating macrolides like azithromycin are bacteriostatic and kill only small subpopulation of cells^61^. Loss of green fluorescence during intracellular macrophage infection might indicate very high AZI levels inside macrophages^62,63^, leading to high mistranslation and/or leakage of bacterial cell contents, such as GFP.

We found that human urine, which is a niche encountered by urinary pathogens, induces translational frameshifting (Fig. 4). Stop codon readthrough levels are slightly increased in urine. As human urine is known to be slightly acidic^64^ the effects on mistranslation might be due to the lower pH, which is known to induce misreading^39^, however increased frameshifting during growth in urine has not been described previously. Some other urine components or the lack thereof, can also change translational fidelity. Increased mistranslation in urine can increase bacterial survival during stress^7,33^ and affect the virulence^13^ of urinary pathogens by altering the proteome.

Laboratory strains were approximately 2 times more prone to both stop codon readthrough and frameshifting than clinical isolates (Fig. 3). Study of *E. coli* natural isolates found a tenfold difference in mistranslation rate between the isolates, however the difference decreased significantly after several generations^2^. It is possible that freshly isolated clinical strains would have a higher variability in translational fidelity.

In standard laboratory conditions, stop-codon readthrough levels were between 0.4 and 2% (Figs. 3 and 4). Standard laboratory media, whether rich or minimal, had little effect on the mistranslation rate. TGA was the most misread stop codon and TAA was the strictest stop codon, which correlates with previously published data^32,38,65^. Most translational misreadings happen due to near-cognate mismatches^30,34,35^. The availability of release factors and near-cognate aa-tRNA molecules affect the stop codon readthrough rates^34^. TAA is the most prevalent stop codon in *E. coli*, which is commonly used in highly expressed genes, and it is recognised by both release factors RF1 and RF2^66^. This might explain why TAA stop is correctly recognised: either one of the release factors is easily available. TGA stop codon is recognised by RF2 alone^66^, which competes with tryptophan and cysteine tRNAs for near-cognate pairing^34^. The nucleotide directly following stop codon also has a minor effect on termination efficiency, as our TGAA sequence is slightly more prone to overreading than TGAT. This is in line with previous findings^67^.

Frameshift rates of reporters in our set remained between 0.3 and 4% in standard conditions (Figs. 3 and 4), and the rates varied a lot between reporters. The highest frameshift rates were detected in T-track FS reporter T7 and A-track A5, which are known slippery sequences^29,68–70^. Interestingly A-track containing FS reporters A6T, which has 6 consecutive adenines, had extremely low, close to detection limit, mistranslation rate. This shows that frameshift rates depend highly on specific genetic sequence and no generalisation can be done for overall frameshifting. However, the developed reporter plasmid system is a great tool to measure frameshift of any sequences of interest with high sensitivity. Our reporters confirm that the levels of both stop-codon readthrough and frameshifting depend also on the time of measurement and growth phase^29^.

Our reporter set showed that stressors, such as antibiotics and growth in urine, did not increase mistranslation in a universal manner, but rather affected only specific sequences. This is in line with previously published data^30,34^. Nonsense reporters with low basal values and frameshift reporters with high basal values are more increased during stress. While a 2-time increase of the TAA stop codon readthrough might seem low, one must keep in mind that TAA is the most used stop codon, so a two-fold increase in readthrough may have detrimental effects on bacterial well-being. On the other hand, each reporter helps to visualize just one type of ribosomal misreading: all these misreadings, including amino acid substitutions, which cannot be detected with these reporters, happen simultaneously, and lead to a significant overall increase of aberrant proteins. This means that tolerable protein mistranslation levels might have been previously underestimated, and the heterogeneous proteomes of individual bacterial cells might have a bigger role in bacterial heterogeneity and survival than previously thought.

### Supplementary Information

Supplementary Information.

## Data Availability

The authors confirm that the data supporting the findings of this study are available within the article and its supplementary materials. Plasmids used in this project will be made available through Addgene (accession numbers 208183…208195).
