# Supplementary material for: Fluorescent reporters give new insights into antibiotics-induced nonsense and frameshift mistranslation
Source: Sci Rep. 2024 Mar 22;14:6883. doi: 10.1038/s41598-024-57597-8 (PMC10959953; doi:10.1038/s41598-024-57597-8)
Supplement: Supplementary file 1 — Supplementary Information. [file 41598_2024_57597_MOESM1_ESM.docx]

**SUPPLEMENTARY DATA**

**FLUORESCENT REPORTERS GIVE NEW INSIGHTS INTO ANTIBIOTICS-INDUCED NONSENSE AND FRAMESHIFT MISTRANSLATION**

Mariliis Hinnu^1,*^, Marta Putrinš^1,2^, Karin Kogermann^2^, Niilo Kaldalu^1^, Tanel Tenson^1^

^1^University of Tartu, Institute of Technology, Tartu 50411, Estonia

^2^University of Tartu, Institute of Pharmacy, Tartu 50411, Estonia

*Corresponding author: [mariliis.hinnu@ut.ee](mailto:mariliis.hinnu%20@ut.ee)

**SUPPLEMENTARY MATERIALS AND METHODS**

*Promoter region optimisation:* different plasmid variants with additional dnaK promoter were constructed with CPEC to increase fluorescence signal. Single colonies of DH5α from CPEC transformation LB-agar plates (with 100 µg/ml ampicillin) were picked and grown aerobically at 37 °C overnight in 3 ml of LB medium containing 100 µg/ml ampicillin. 100 µl of overnight culture was added to 96-well plate and OD_600_ and green fluorescence was measured with Synergy Mx platereader (λ_ex_ 485/9 nm/λ_em_ 510/9 nm, gain 100).

*mScarlet variants platereader experiments:* Mid-exponential cultures (OD_600_ ~0.4) in MHB (cation-adjusted) were diluted to OD_600_ 0.025 in 100 µl volume on a 96-well plate and incubated at 37 °C with continuous shaking with Synergy Mx platereader. OD_600_, green fluorescence (λ_ex_ 485/9 nm/λ_em_ 510/9 nm, gain 80) and red fluorescence (λ_ex_ 569/13.5 nm/λ_em_ 600/17 nm, gain 100) were recorded every 15 minutes.

*Aminoglycosides:* Stock solutions of 25 mg/ml of apramycin sulfate (Sigma), streptomycin sulfate (Fluka BioChemica), tobramycin (Sigma), kanamycin sulfate (BioChemica Applichem) were prepared in Milli-Q water and stored at 4 °C. Gentamicin 50 mg/ml smart media solution (Naxo) was used.

*Autofluorescence measurements by flow cytometry:* Fluorescence data of *E. coli* MG1655 with non-fluorescent plasmid pBR322 was recorded with flow cytometry (BD LSRFortessa, BD Biosciences) after 24 h of growth in LB medium in the presence of aminoglycosides. Geometric means of cells gated according to forward and side scatter plots were analysed.

*MG1655 WT and MG1655 ΔL31 platereader experiment:* 20% stock solution of Bacto™ Casamino Acids (BD, USA) (CAA) was prepared in distilled H_2_O and filter-sterilized. The solution was stored at +4 °C up to 1 week. 0.2% final concentration of CAA was used in M9 minimal medium containing 0.2% glucose. Precultures were started from DMSO stocks and grown in 3 ml of M9 minimal medium with glucose to exponential phase and diluted to OD*_600_* 0.05 in indicated medium. 50 µl of the diluted culture was added to 50 µl of indicated medium and incubated aerobically in a microtiter platereader (Biotek Synergy Mx).

*MG1655 WT and MG1655 ΔL31 timepoints experiment:* Exponential precultures in 3 ml of LB were diluted to OD_600_ 0.05 in 10 ml of filtered LB medium in 100 ml flasks. Cultures were grown aerobically at 37 °C. 150 µl samples were taken in each timepoint and put on ice. Samples were stored in 15% glycerol at ­­­80 °C until flow cytometry analysis.

*Bicarbonate MHB medium:* 1M sodium bicarbonate [NaHCO3 (POCH, Poland)] stock solution was prepared in distilled H_2_O and filter-sterilized. The solution was stored at +4 °C. 25 mM final concentration of bicarbonate was added to sterile cation-adjusted MHB medium and the pH was adjusted to 7.4 with 0.1M HCl at the start of the experiment. As the pH of unbuffered medium with bicarbonate gets alkaline within the first few hours^1^, experiments with bicarbonate-containing media were not included in the main article.

*E. coli vs Salmonella mistranslation: Salmonella enterica* serovar Typhimurium SL1344 (streptomycin resistant) was compared with *E. coli* CFT073. Mistranslation plasmids were transformed into the *Salmonella* strain via electroporation. Bacteria with plasmids were streaked from glycerol stocks onto LB-agar plates with selection antibiotics and incubated overnight at 37 °C. Single colonies were resuspended in 1 ml of sterile 1X PBS. 10 µl of the bacterial suspension was added to 90 µl of growth medium (MHB with 25 mM bicarbonate) with selection antibiotics in a 96-well plate and incubated aerobically at 37 °C overnight in a plate-shaking incubator. Next day the overnight culture was diluted 100X into fresh medium without antibiotics and incubated in the same conditions for 3 hours. Then 10 µl of preculture was added to 90 µl of fresh medium and incubated at 37 °C in Biotek Synergy H4 with medium shaking for 18 hours.

*Azithromycin timepoints experiment:* Experimental procedure as in the main text, except that experiments were done in MHB medium with bicarbonate. The addition of bicarbonate to growth media increases bacterial sensitivity to azithromycin due to pH increase^1^. The pattern of mistranslation induction by azithromycin is still clearly visible throughout different timepoints and are comparable to those without bicarbonate.

*Aminoglycoside mistranslation:* Experiments were carried out as with amikacin, described in the main article.

*Microscopy image analysis:* Images were analyzed using MicrobeJ^2^. Cells were located on the phase contrast image, green and red fluorescence intensity values were acquired from respective channels. Bacterial cell detections were manually checked and corrected, when necessary. On average about 1400 cells were analyzed in each sample.

*Conditional MICs:* MICs were determined by relevant experimental procedure, i.e medium and inoculum prepared as in the experiment, not by standard MIC protocol^3^. The inoculum was therefore higher than in the standard MIC assay. The conditional MIC plates were incubated as in the mistranslation experiment – with shaking. According to our experience shaking can also affect the MIC. The MICs were determined after 18 – 20 h of incubation by visual inspection.

*Macrophage infection and microscopy*. Uropathogenic *E. coli* CFT073 carrying T7 frameshift reporter plasmid was used to infect mouse macrophage-like J774 cells according to protocol^10^ with minor modifications. Namely, bacterial cells from a colony grown overnight (<20 h) on LB-agar (containing 100 µg/ml carbenicillin) were used for infecting the macrophages. Multiplicity of infection was 50 bacterial cells per 1 macrophage (MOI-50). Bacteria were added to 96-well microscopy cell culture plates, centrifuged, and allowed to phagocytize for 1 h. To remove non-phagocytized bacteria cell culture medium (RPMI 1640 supplemented with 10% fetal bovine serum) was removed and replaced with same medium containing 100 µg/ml gentamicin for 1 h, after which medium was replaced with medium containing final concentration of 5 µg/ml gentamicin and serial dilutions of AZI. Plates were incubated overnight in a 5% CO2 incubator. Next day cells were fixed in 4% formaldehyde in 1X PBS for 20 min. Fixation solution was removed, cells were washed, and cells were stained for 15 min with 100 ng/ml DAPI in 1X PBS containing 0.1% Triton X. Staining solution was removed and cells washed thrice with 1X PBS, after which left in 1X PBS. Cells were imaged with Zeiss LSM900 confocal microscopy system with 20X magnification. Green (Ex 488 nm: 1.8%; Em 500-560 nm), red (Ex 561 nm: 2.8%; Em 576-700 nm), and blue (Ex: 405 nm: 3.4%; Em 400-500 nm) fluorescence was recorded. Fluorescence levels were adjusted during image processing for presentation purposes in an equal manner.

**Supplementary references:**

1. Hinnu, M., Putrinš, M., Kogermann, K., Bumann, D., & Tenson, T. (2022). Making Antimicrobial Susceptibility Testing More Physiologically Relevant with Bicarbonate? *Antimicrobial Agents and Chemotherapy*, *66*(5). <https://doi.org/10.1128/AAC.02412-21>
2. Ducret, A., Quardokus, E. M., & Brun, Y. V. (2016). MicrobeJ, a tool for high throughput bacterial cell detection and quantitative analysis. *Nature Microbiology*, *1*(7), 1–7. <https://doi.org/10.1038/nmicrobiol.2016.77>
3. EUCAST. (2003). Determination of minimum inhibitory concentrations (MICs) of antibacterial agents by broth dilution. *Clinical Microbiology and Infection*, *9*(8), ix–xv. <https://doi.org/10.1046/j.1469-0691.2003.00790.x>
4. Hayashi, K., Morooka, N., Yamamoto, Y., Fujita, K., Isono, K., Choi, S., Ohtsubo, E., Baba, T., Wanner, B. L., Mori, H., & Horiuchi, T. (2006). Highly accurate genome sequences of Escherichia coli K-12 strains MG1655 and W3110. *Molecular Systems Biology*, *2*(1), 2006.0007. <https://doi.org/10.1038/MSB4100049>
5. Lilleorg, S., Reier, K., Remme, J., & Liiv, A. (2017). The Intersubunit Bridge B1b of the Bacterial Ribosome Facilitates Initiation of Protein Synthesis and Maintenance of Translational Fidelity. *Journal of Molecular Biology*, *429*(7), 1067–1080. <https://doi.org/10.1016/J.JMB.2017.02.015>
6. Datsenko, K. A., & Wanner, B. L. (2000). One-step inactivation of chromosomal genes in Escherichia coli K-12 using PCR products. *Proceedings of the National Academy of Sciences*, *97*(12), 6640–6645. <https://doi.org/10.1073/pnas.120163297>
7. Mobley, H. L., Green, D. M., Trifillis, A. L., Johnson, D. E., Chippendale, G. R., Lockatell, C. V., Jones, B. D., & Warren, J. W. (1990). Pyelonephritogenic Escherichia coli and killing of cultured human renal proximal tubular epithelial cells: Role of hemolysin in some strains. *Infection and Immunity*, *58*(5), 1281–1289. <https://doi.org/10.1128/IAI.58.5.1281-1289.1990>
8. Agarwal, D., Kamath, D., Gregory, S. T., & O’Connor, M. (2015). Modulation of Decoding Fidelity by Ribosomal Proteins S4 and S5. *Journal of Bacteriology*, *197*(6), 1017–1025. <https://doi.org/10.1128/jb.02485-14>
9. Agarwal, D., Gregory, S. T., & O’Connor, M. (2011). Error-Prone and Error-Restrictive Mutations Affecting Ribosomal Protein S12. *Journal of Molecular Biology*, *410*(1), 1–9. <https://doi.org/10.1016/j.jmb.2011.04.068>
10. Kerkez, I., Tulkens, P. M., Tenson, T., Van Bambeke, F., & Putrinš, M. (2021). Uropathogenic Escherichia coli Shows Antibiotic Tolerance and Growth Heterogeneity in an In Vitro Model of Intracellular Infection. *Antimicrobial Agents and Chemotherapy*, *65*(12), 10.1128/aac.01468-21. <https://doi.org/10.1128/aac.01468-21>


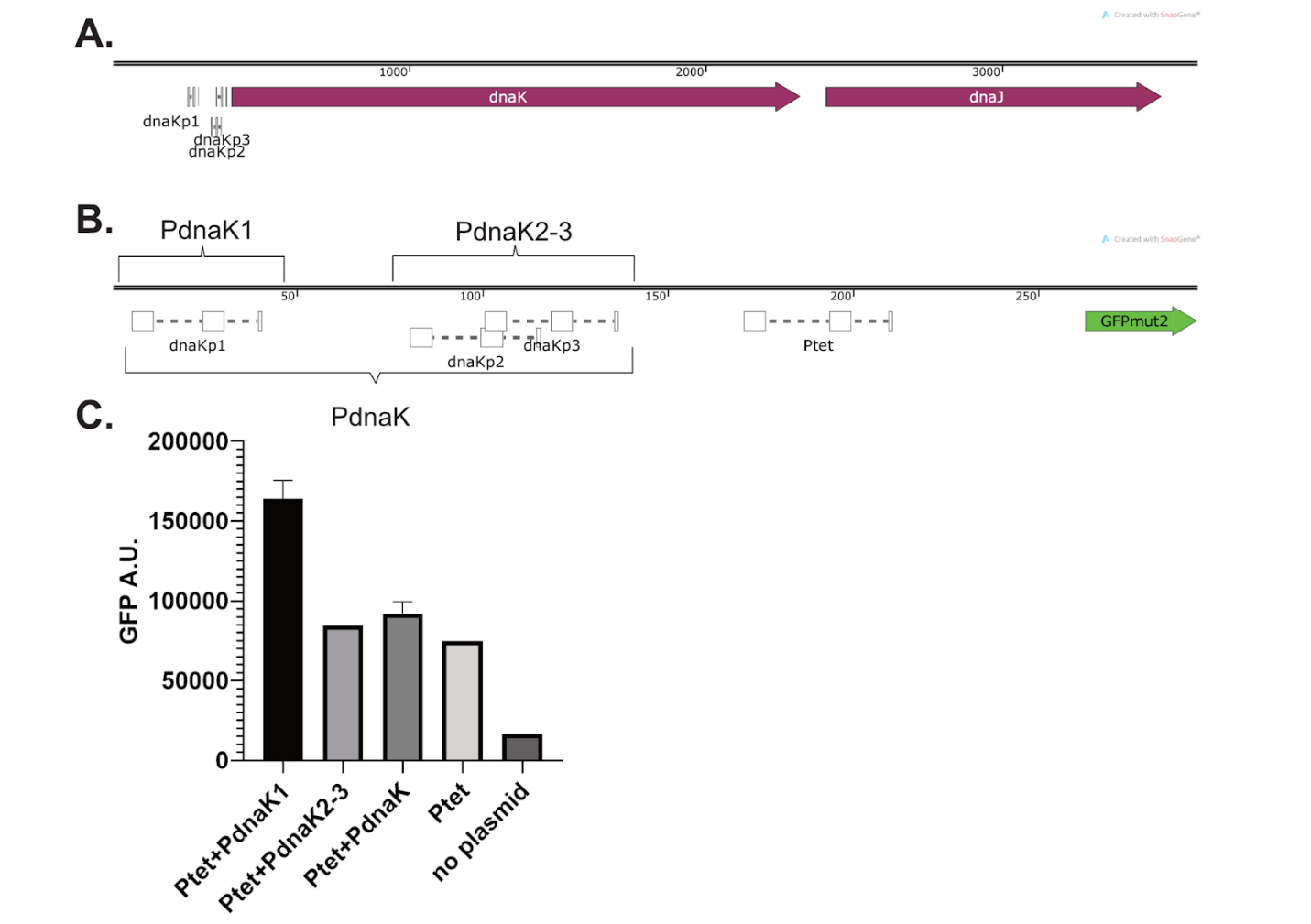


Figure S1. An additional stress-inducible dnaK promoter was inserted in front of the reporter sequence in order to increase expression levels of reporter proteins. A. The native heat shock protein chaperon DnaK operon in E. coli MG1655 genome (NCBI Accession number NC_000913). B. Promoter regions of the mistranslation reporter plasmids. dnaK promoter variants were cloned in front of the constitutive tet-promoter transcribing both GFP and mScarlet‑I in order to increase expression levels of fluorescent proteins. C. dnaK promoter 1 gave the highest GFP level in DH5α overnight cultures. Fluorescence of overnight bulk cultures were measured using a platereader. Fluorescence was normalized according to OD_600_.


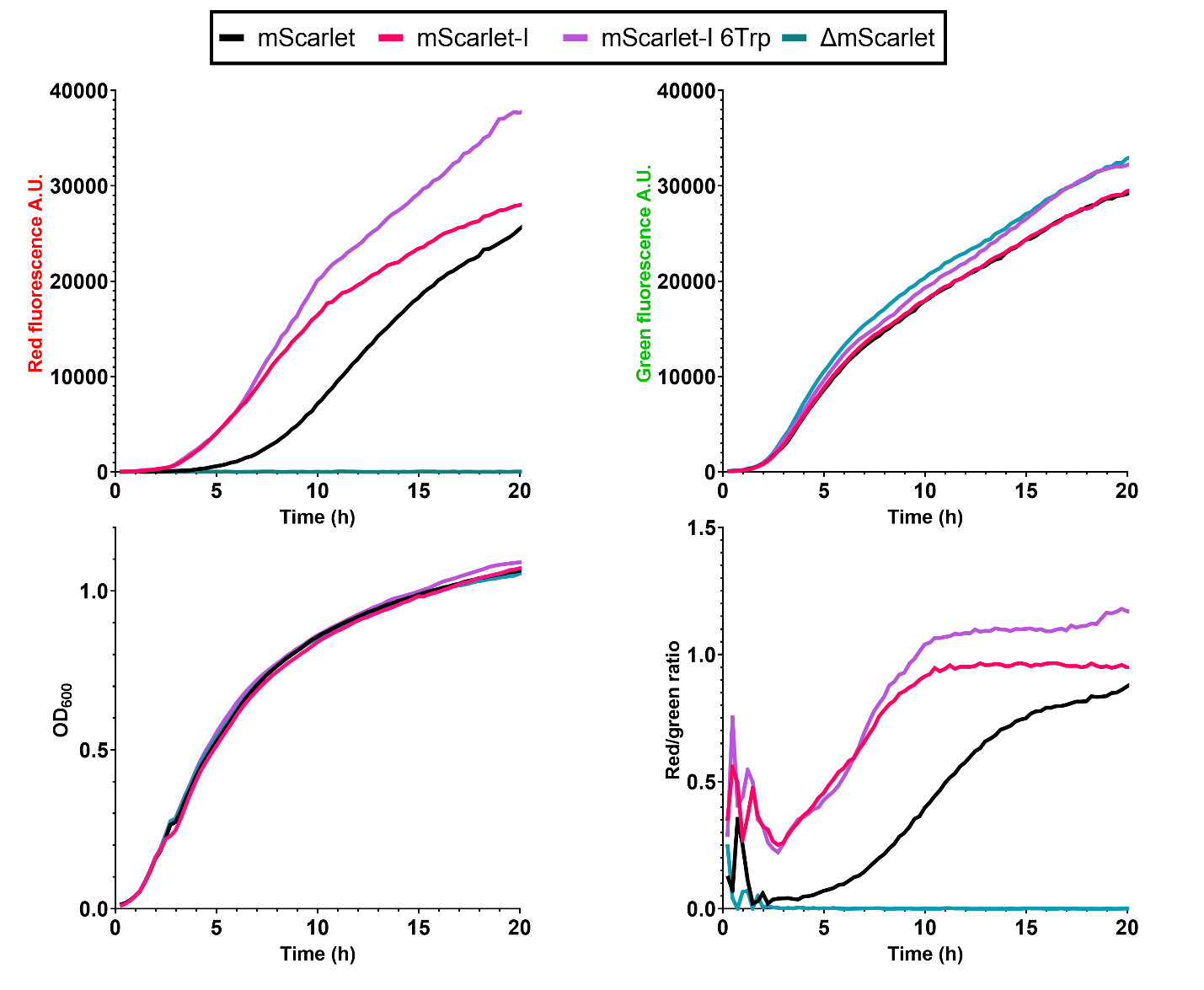


Figure S2. Mistranslation reporters’ fluorescence dynamics of E. coli MG1655 bulk cultures. mScarlet-I was chosen as the reporter protein, as it is a bright red fluorescent protein (RFP), which has much faster maturation time than other RFPs, being more similar to that of GFP. No autofluorescence or GFP signal bleedthrough was detected in the mScarlet spectrum, as shown by ΔmScarlet reporter.  A 6 bp insertion at the sites where stop-codons or frameshift mutations were introduced for mistranslation reporters does not hamper the fluorescence signal – mScarlet‑I 6Trp, which has tryptophan and serine codons at positions 6 and 7, respectively, has high red fluorescence. However, the mutation can possibly affect the stability of mRNA, as is indicated by the simultaneous increase of both red and green fluorescence signal of the same reporter. Platereader growth experiment data in MHB medium.


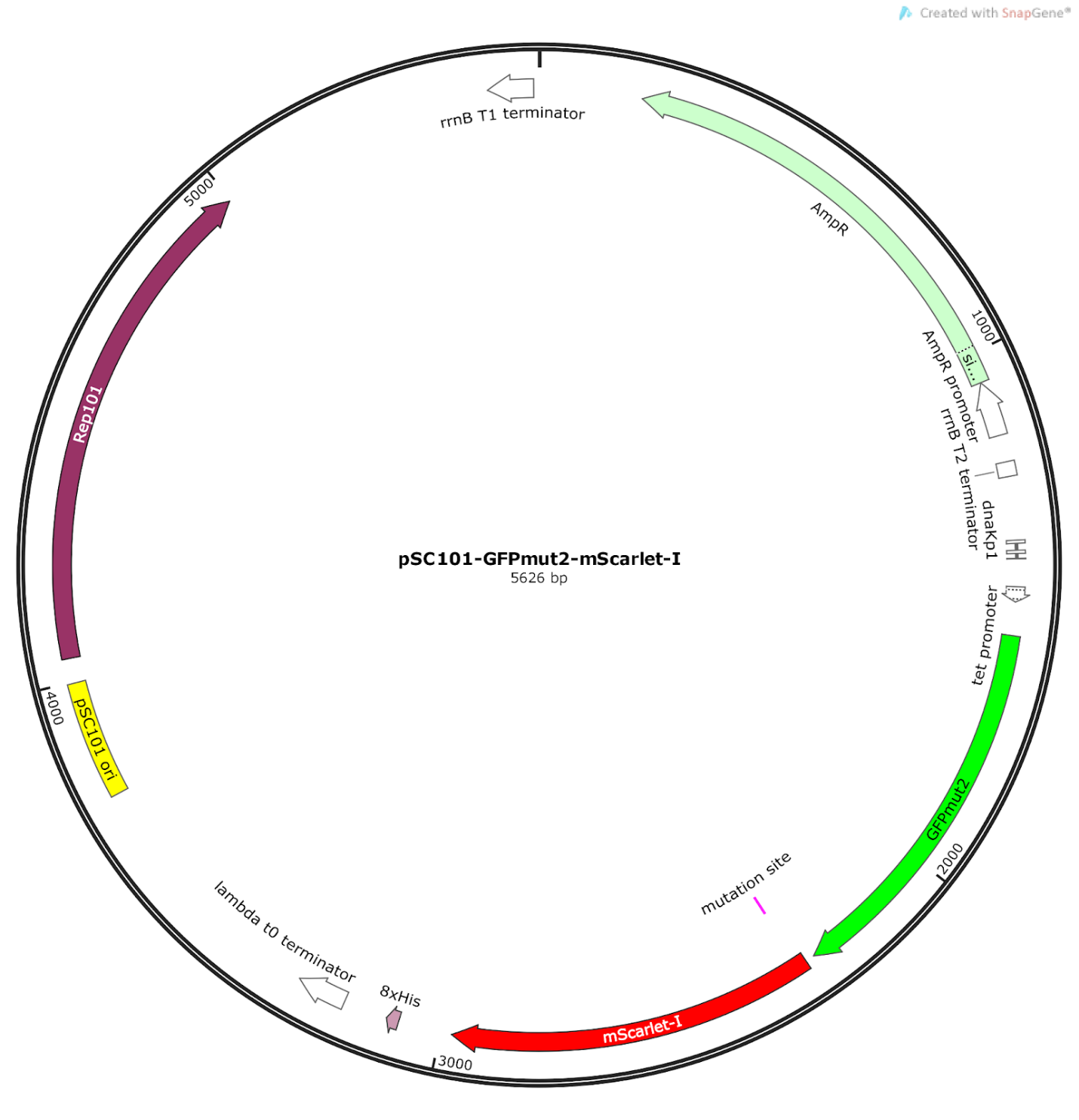


Figure S3. Plasmid map of the mistranslation reporter plasmid. 6-7 nt were inserted into the 6th codon of the mScarlet-I gene via primer design to introduce stop codons of frameshift mutations. Both GFPmut2 and mScarlet-I are transcribed on a single mRNA. The distance between the two genes is 17 bp. mScarlet-I has its own ribosomal binding site.


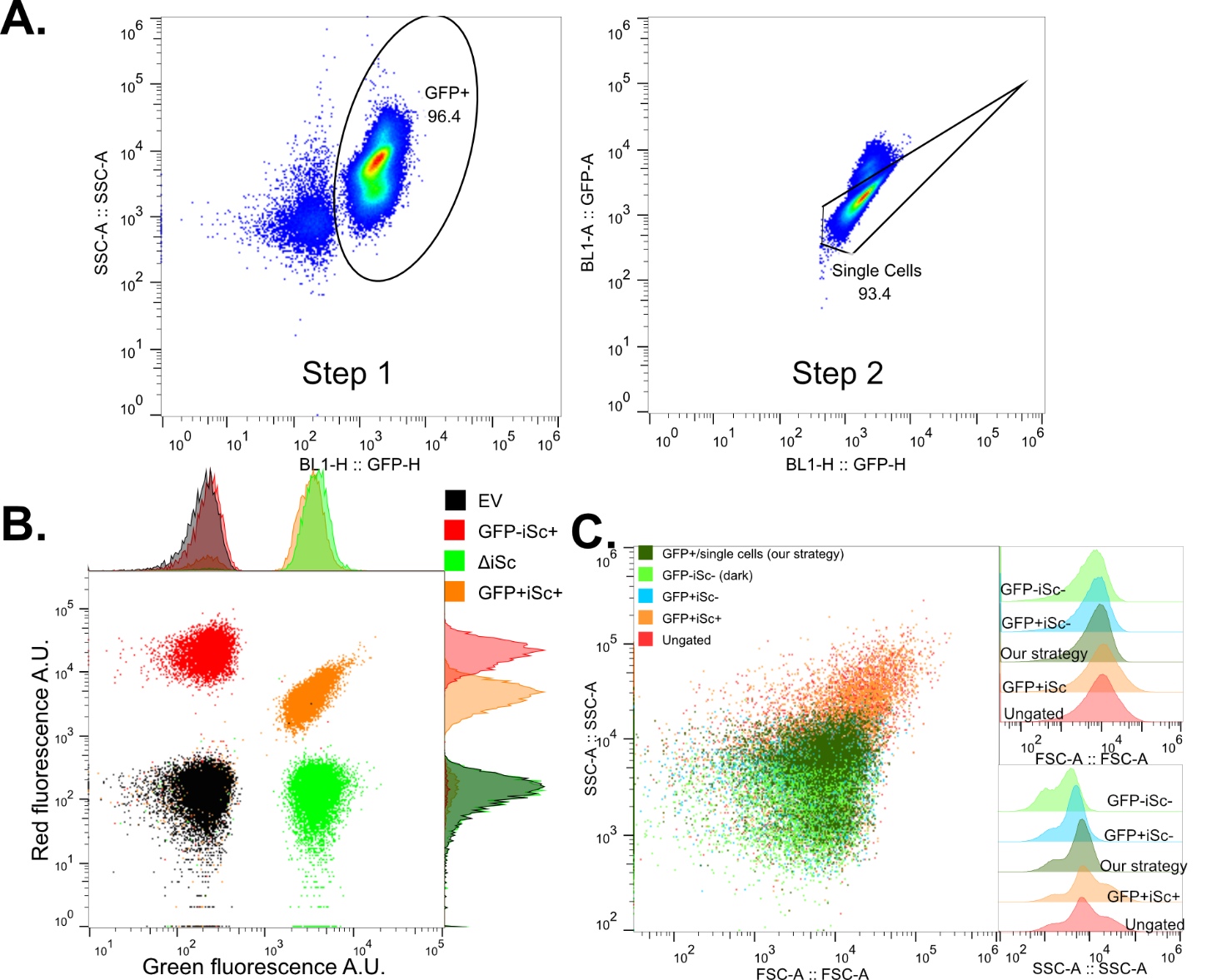


Figure S4. A. Gating strategy of flow cytometry data. First green fluorescent cells (GFP+) are gated from side scatter (SSC-A) and green fluorescence (BL1-H :: GFP-H) plots. Then single cell population is chosen from green fluorescence height (BL1-H :: GFP-H) and green fluorescence area (BL1-H :: GFP-A) plots. CFT073 GFP+iSc+ cells after 4 h in MHB medium used as an example. B. MG1655 with different control plasmids carrying either empty vector plasmid (EV), GFP-negative/iSc-positive (GFP-iSc+), iSc-negative/GFP-positive (ΔiSc) or GFP-positive/iSc-positive (GFP+iSc+). Cells gated according to forward and side scatter only. EV cells overlap with noise, small population of which is also present in fluorescent cell samples gated with this strategy. There is no fluorescence spillover of GFP into iSc channel, and minimal spillover from iSc to GFP channel. No compensation was therefore enforced. Cells grown in LB medium for 18h. C. Forward (FSC) and side scatter (SSC) plots of CFT073 GFP+iSc+ (positive control) cells treated with 8 µg/ml of azithromycin for 4 h in MHB. Bacterial cells cannot be distinguished from noise according to forward and side scatter.

**
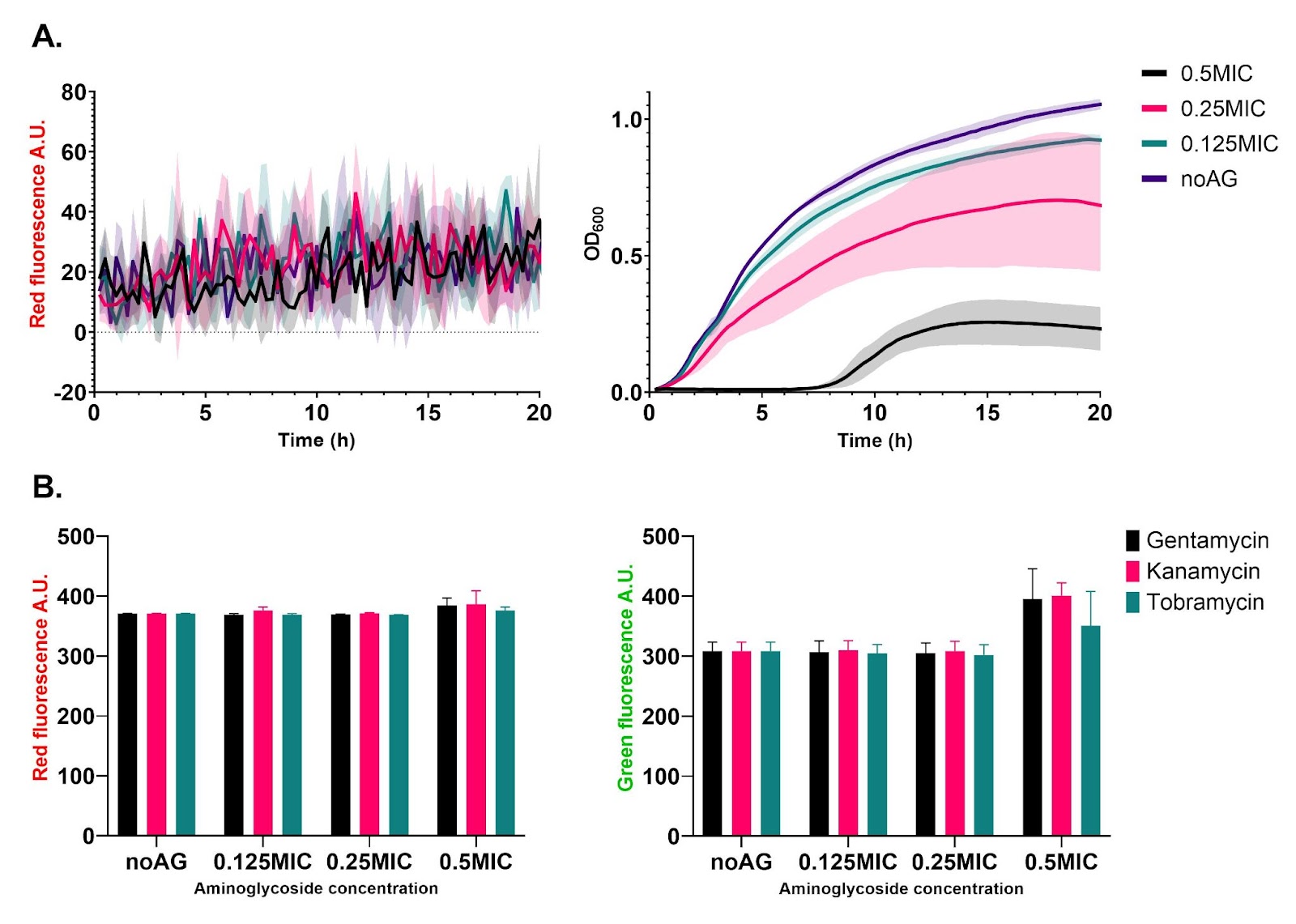
**

Figure S5. Autofluorescence can be affected by antibiotic-induced stress. A. Red autofluorescence and growth of MG1655 with red negative pSC101-GFPmut2-ΔmScarlet plasmid at the presence of subinhibitory kanamycin concentrations in MHB medium. Red autofluorescence of bulk cultures is not significantly affected. Data represent means and standard deviations of at least 3 replicates from platereader data at the spectrum of mScarlet-I. B. Red and green autofluorescence of MG1655 cells with non-fluorescent pBR322 plasmid. Red autofluorescence remains relatively unaffected, but green autofluorescence increased with all tested aminoglycosides at the highest tested antibiotic concentration. Flow cytometry data after 24 h of growth in LB medium. Mean±SD; N=2.
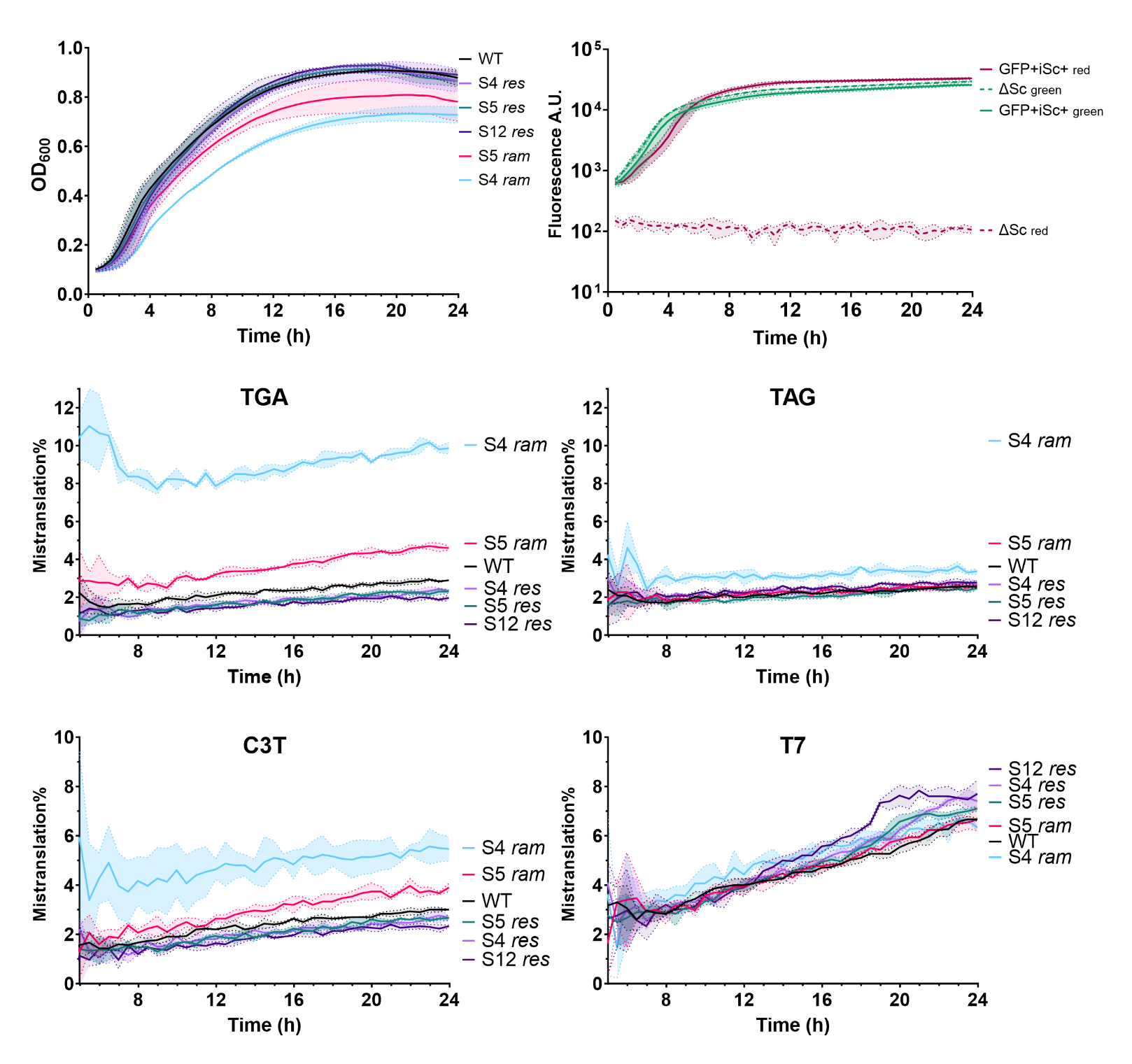


Figure S6. Platereader data of MC361 ram and res mutants. Top left: Growth curves of the strains with GFP+iSc+ (positive control) plasmid. Top right: Green and red fluorescence levels of WT with GFP+iSc+ (positive control) or ΔSc (negative control) plasmid. Mistranslation levels over time of TGA and TAG nonsense reporters, and C3T and T7 frameshift reporters. Data shown from 5 hours since the start of the experiment, as reporter signal cannot be correctly determined in earlier timepoints by the platereader. Means ± SD (3 biological replicates).

**
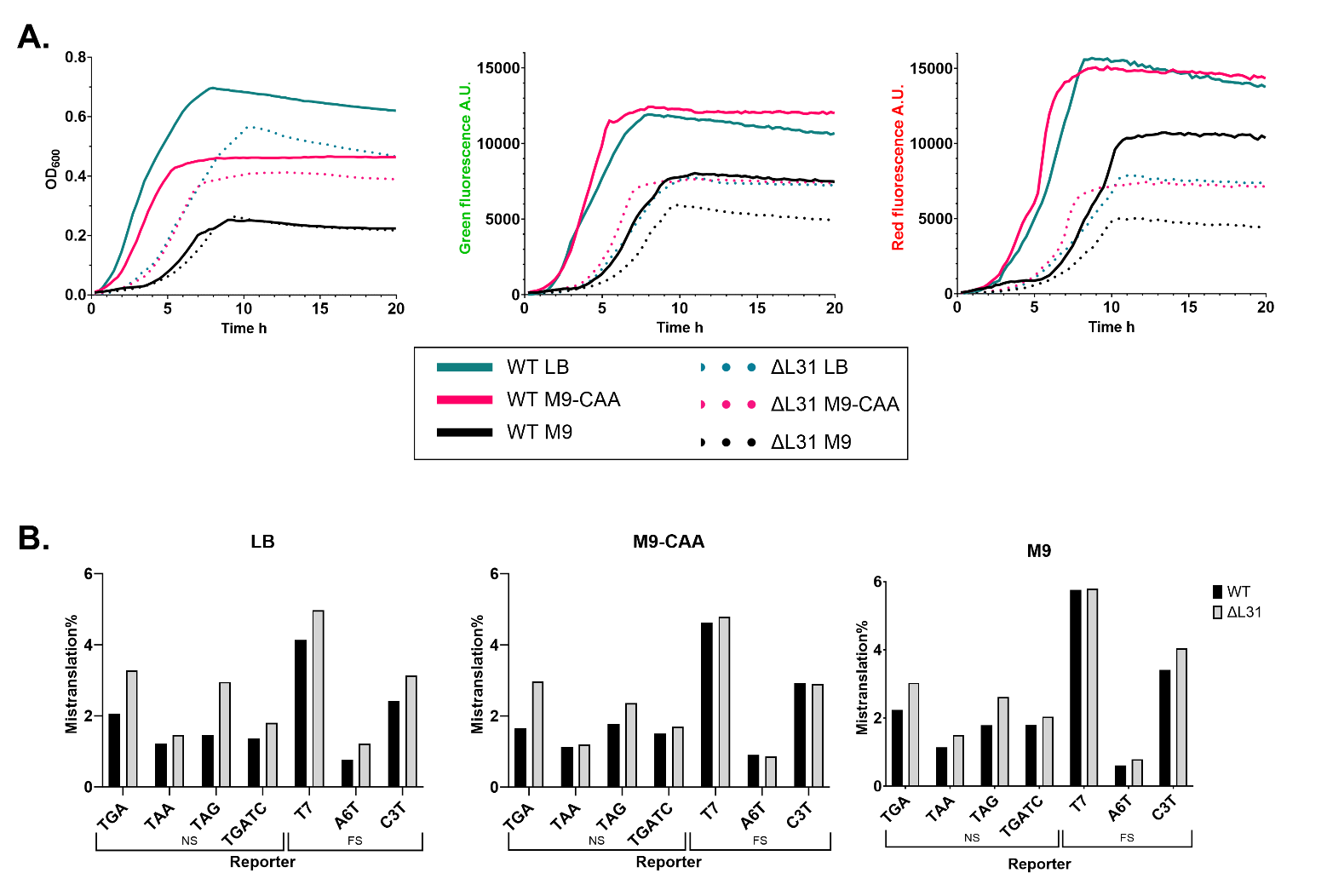
**

Figure S7. Comparison of MG1655 wild-type (WT) and ribosomal ambiguity mutant ΔL31 in 3 different growth media. M9 is supplemented with 0.2% glucose and where indicated additionally with 0.2% casamino acids (CAA). A. The ΔL31 strain with positive control plasmid GFP+iSc+ reveals a longer lag-phase and a lower final optical density in media with amino acids compared to the WT. In addition, the levels of both green and red fluorescent proteins are lower for the mutant. Platereader data. B. Reporter mistranslation signals of the ribosomal ambiguity mutant ΔL31 do not show any significant increase after 20 h incubation at 37 °C. The biggest differences can be seen in LB medium. Data based on bulk culture analysis from a platereader.


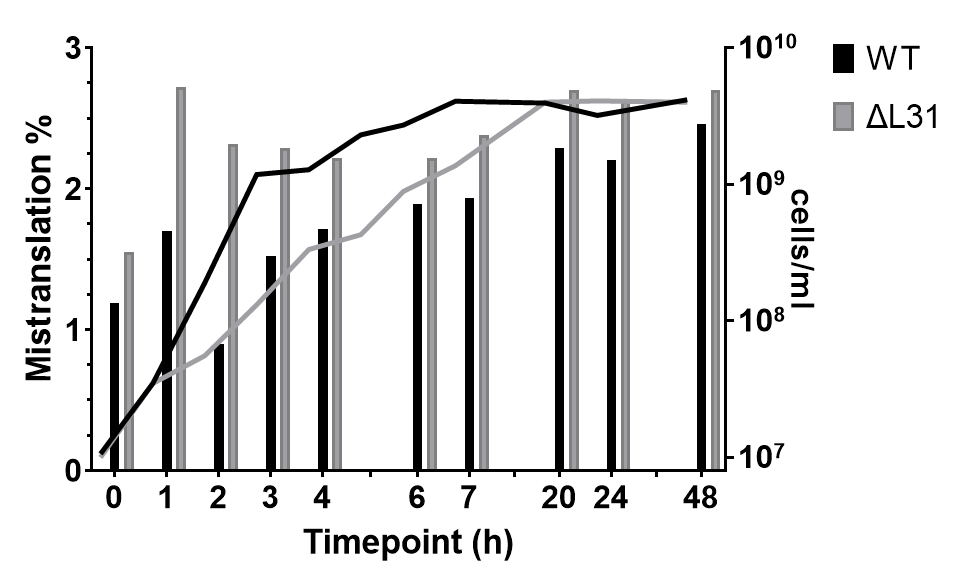


Figure S8. Reporter timepoint optimization using the ribosomal ambiguity mutant ΔL31. Flow cytometry analysis of MG1655 WT and ΔL31 with the mScarlet-I TGA reporter hinted that mistranslation difference between the two strains is the largest within the first few hours of growth.

Figure S9. Growth curves of tested E. coli strains with G+S+ positive control plasmid in MHB medium. All strains have similar growth rate, except for laboratory strain DH5α, which is growing slightly slower. Platereader data. N≥2.

Figure S10. E. coli and Salmonella mistranslation differences were found insignificant. Data after 18 h of growth in MHB with bicarbonate, platereader data.
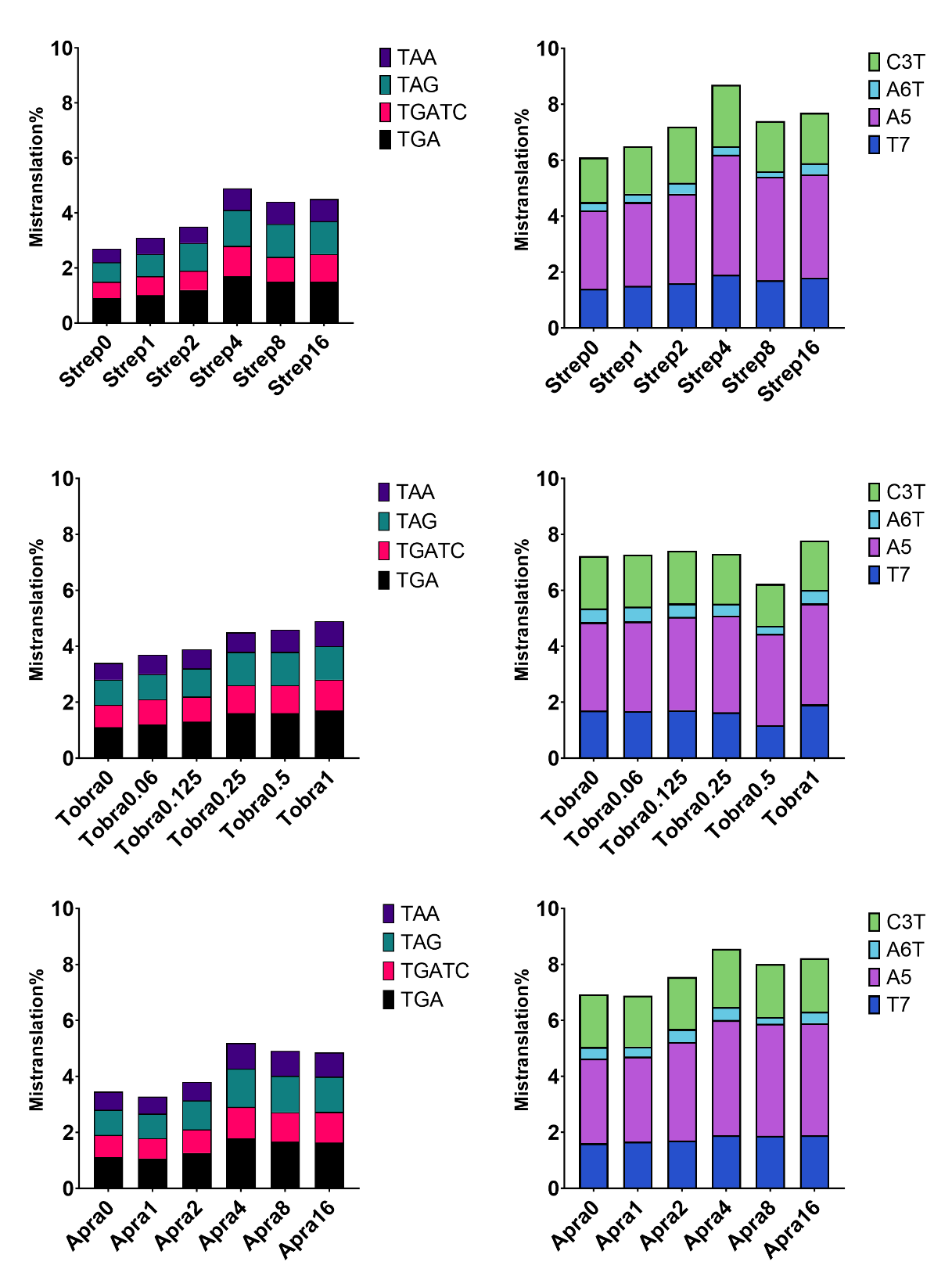


Figure S11. MG1655 WT mistranslation increase with aminoglycosides. All tested aminoglycosides increase stop codon readthrough (left column). Frameshifting (right column) is affected less. Strep = streptomycin, Tobra = tobramycin, Apra = apramycin. Number following the antibiotic abbreviation indicates concentration in µg/ml. Concentration range is equal to 2…1/8 MICs in given conditions. Flow cytometry analysis after 4 h of treatment with antibiotic in M9 minimal medium with 0.2% glucose.


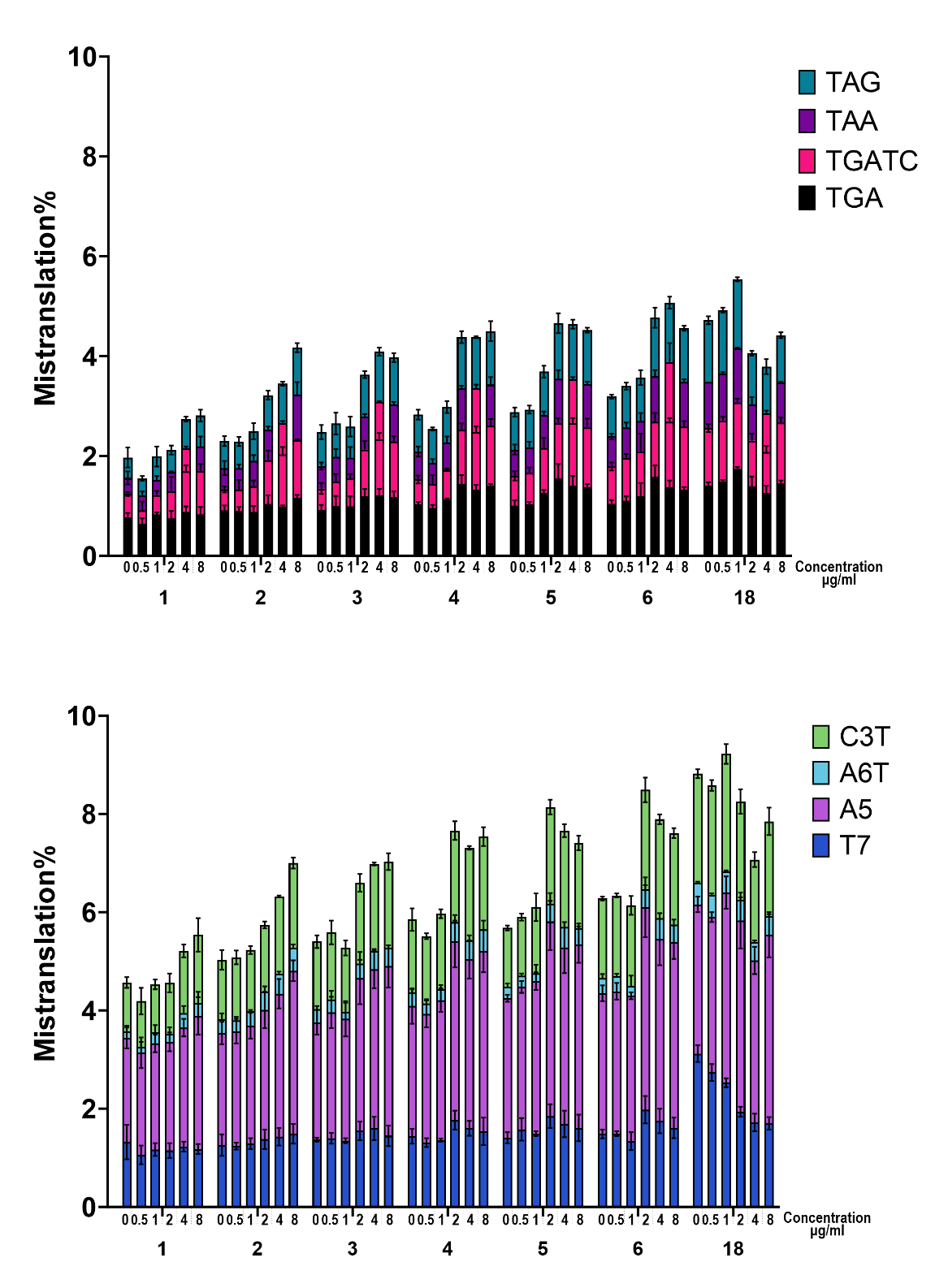


Figure S12. Time-course of mistranslation induction by amikacin (AMI). MG1655 was grown in M9 minimal medium supplemented with 0.2% glucose, treated with AMI at different concentrations, and analyzed by flow cytometry. Mistranslation reporter signal correlates with amikacin concentration within the first few hours of antibiotic treatment. AMI induces stop codon readthrough more than frameshift. Overall frameshift levels are higher. Data represent mean and standard deviations (N=3; mean±SD).


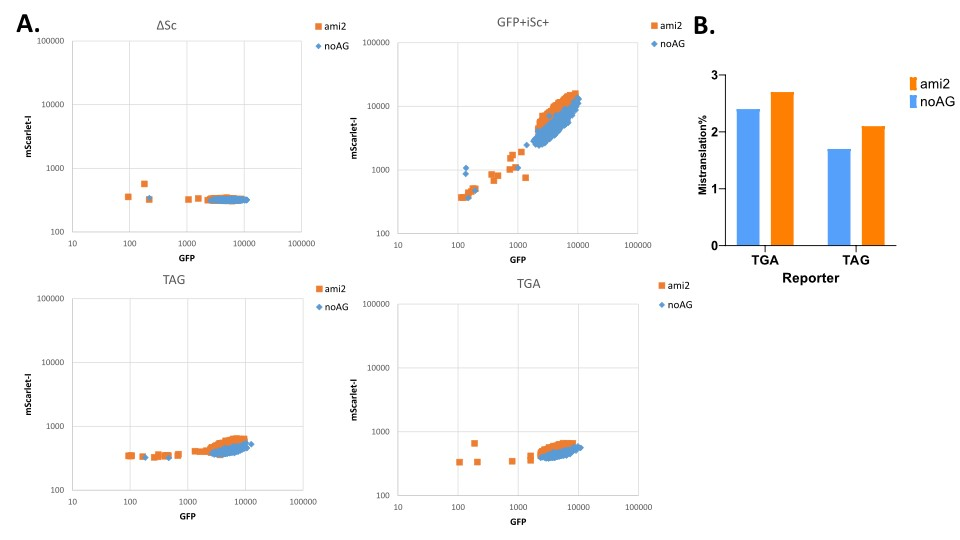


Figure S13. Microscopy image analysis of MG1655 WT nonsense reporters using MicrobeJ plugin for ImageJ. A. Green and red fluorescence mean intensity values of individual cells were plotted for controls and two nonsense reporters. Autofluorescence in mScarlet-I does not increase, as shown by the negative control ΔSc. A slight increase of red signal can be seen with all mScarlet-I reporters, even the GFP+iSc+ positive control that contains intact mScarlet-I. B. Mistranslation was calculated from microscopy data. This method detects higher levels of mistranslation than flow cytometry, however the increase of mistranslation with ½ MIC amikacin is in a similar range.


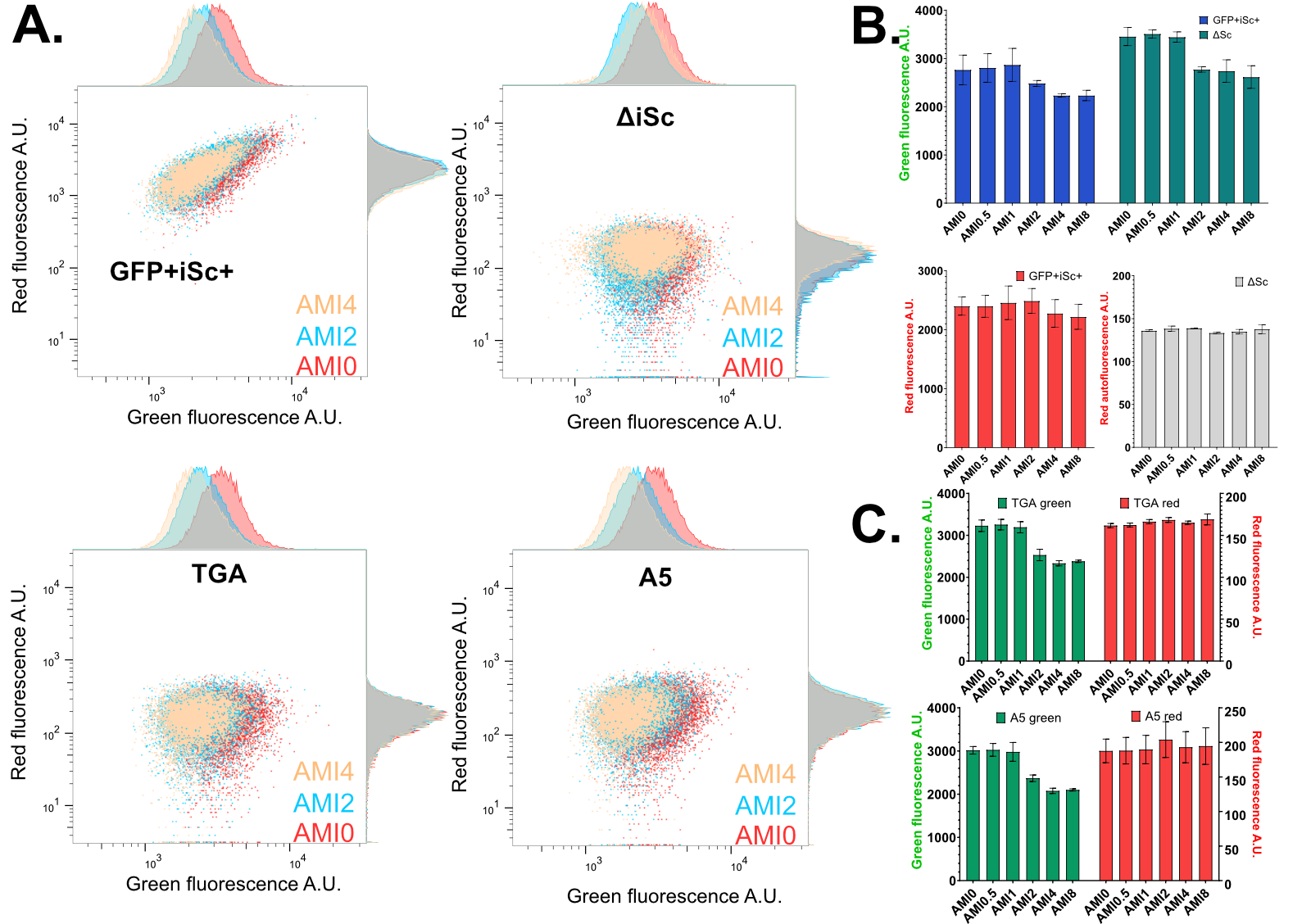


Figure S14. Flow cytometry analysis of MG1655 cells in the presence of amikacin (AMI) after 4h in M9 minimal medium. A. Green and red fluorescence dotplots (gated according to our gating strategy) show that cells form a single subpopulation. Increasing AMI concentrations mainly decrease in green fluorescence. Representative of positive (GFP+iSc-) and negative (ΔiSc) control, TGA nonsense and A5 frameshift reporter plots. B. Geometric means of red or green fluorescence of gated control samples in the presence of AMI. C. Geometric means of red or green fluorescence of gated TGA or A5 samples in the presence of AMI. Red autofluorescence has not been subtracted. B./C. Mean±SD (N=3).


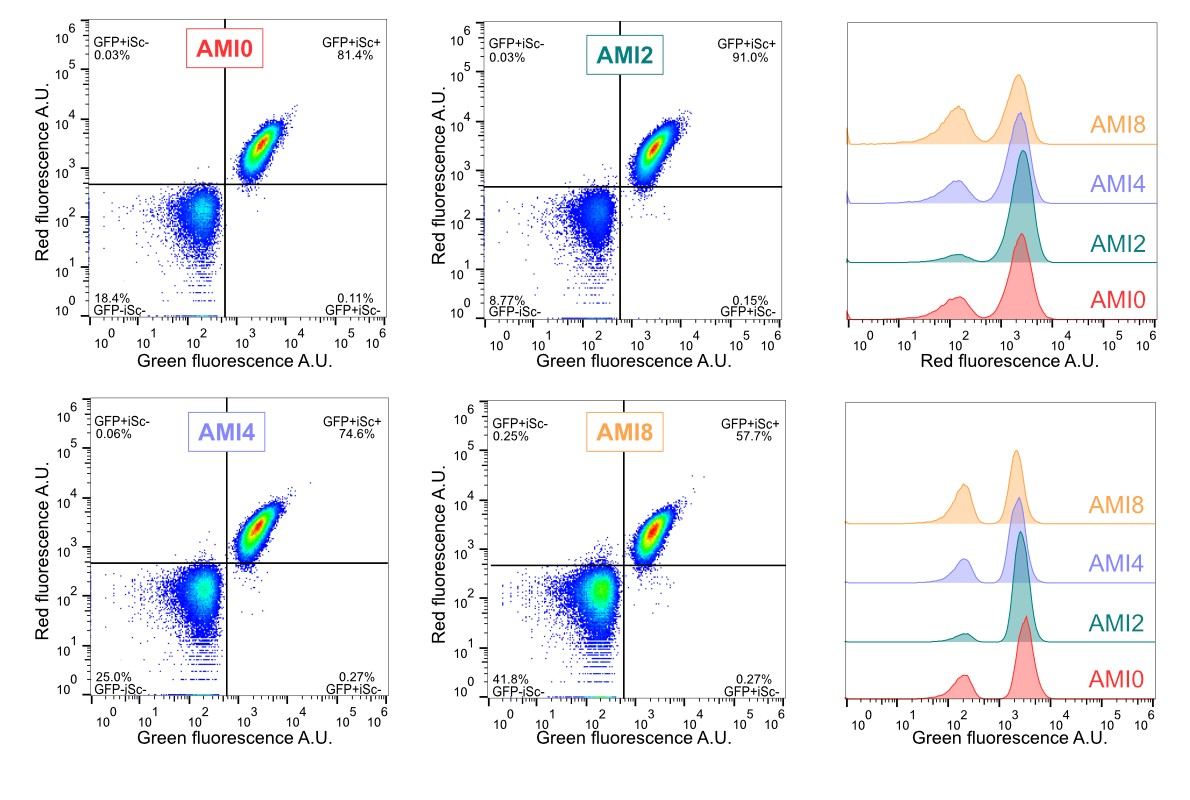


Figure S15. Flow cytometry analysis of GFP+mScarlet-I+ positive control shows an increase of a non-fluorescent population with increasing amikacin (AMI) concentrations. The green and red fluorescence plots shown are ungated, therefore the non-fluorescent population also includes flow cytometry noise. Only green fluorescent populations were chosen for further analysis according to gating strategy shown on Figure S4A. The number after AMI indicates the concentration of the drug in µg/ml.


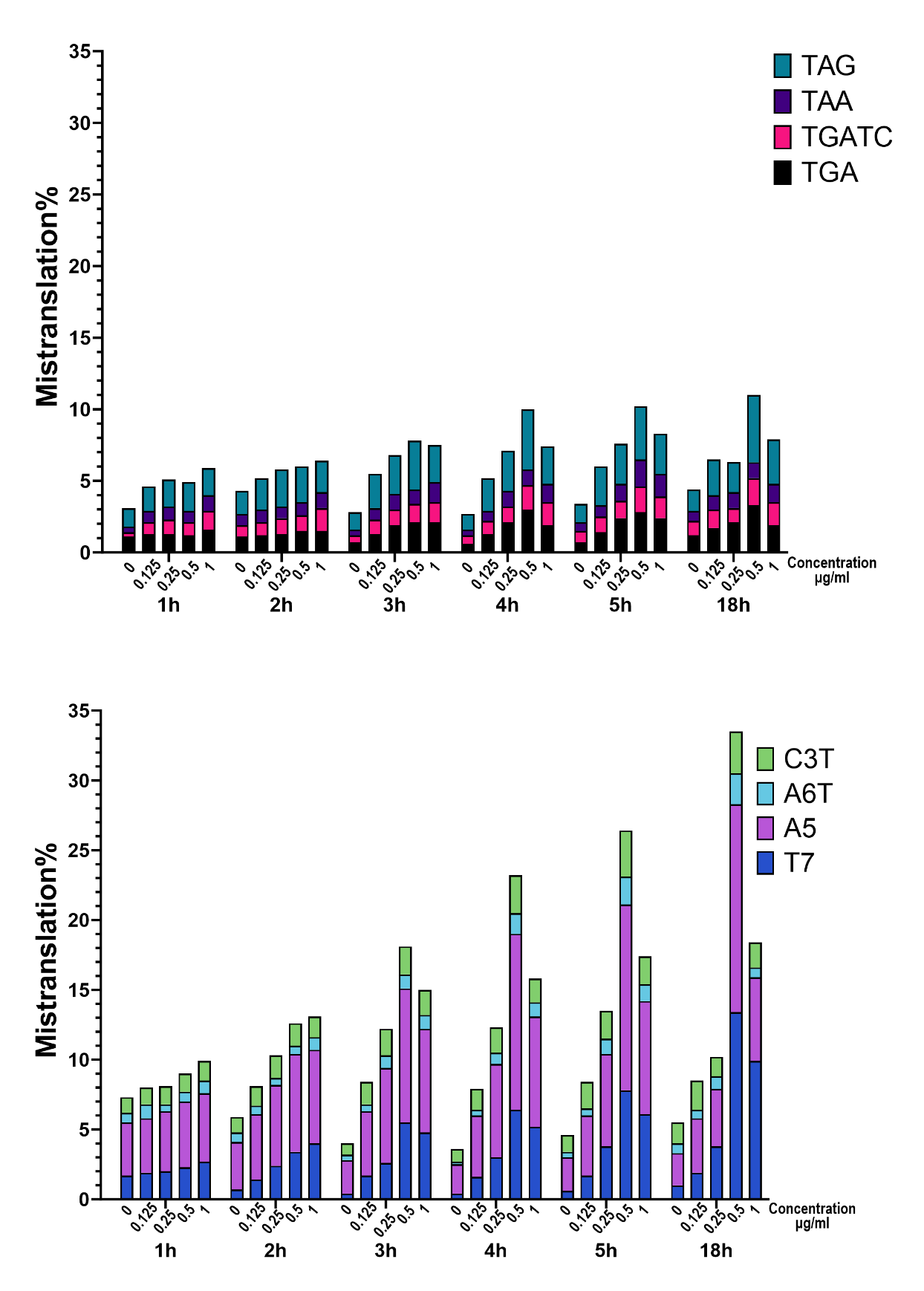


Figure S16. Mistranslation of CFT073 during azithromycin treatment. Azithromycin induces both stop codon readthrough and frameshifting. The highest induction of mistranslation was detected at ½ MIC of azithromycin (0.5 µg/ml). MHB medium (cation-adjusted) with 25 mM bicarbonate, flow cytometry analysis.


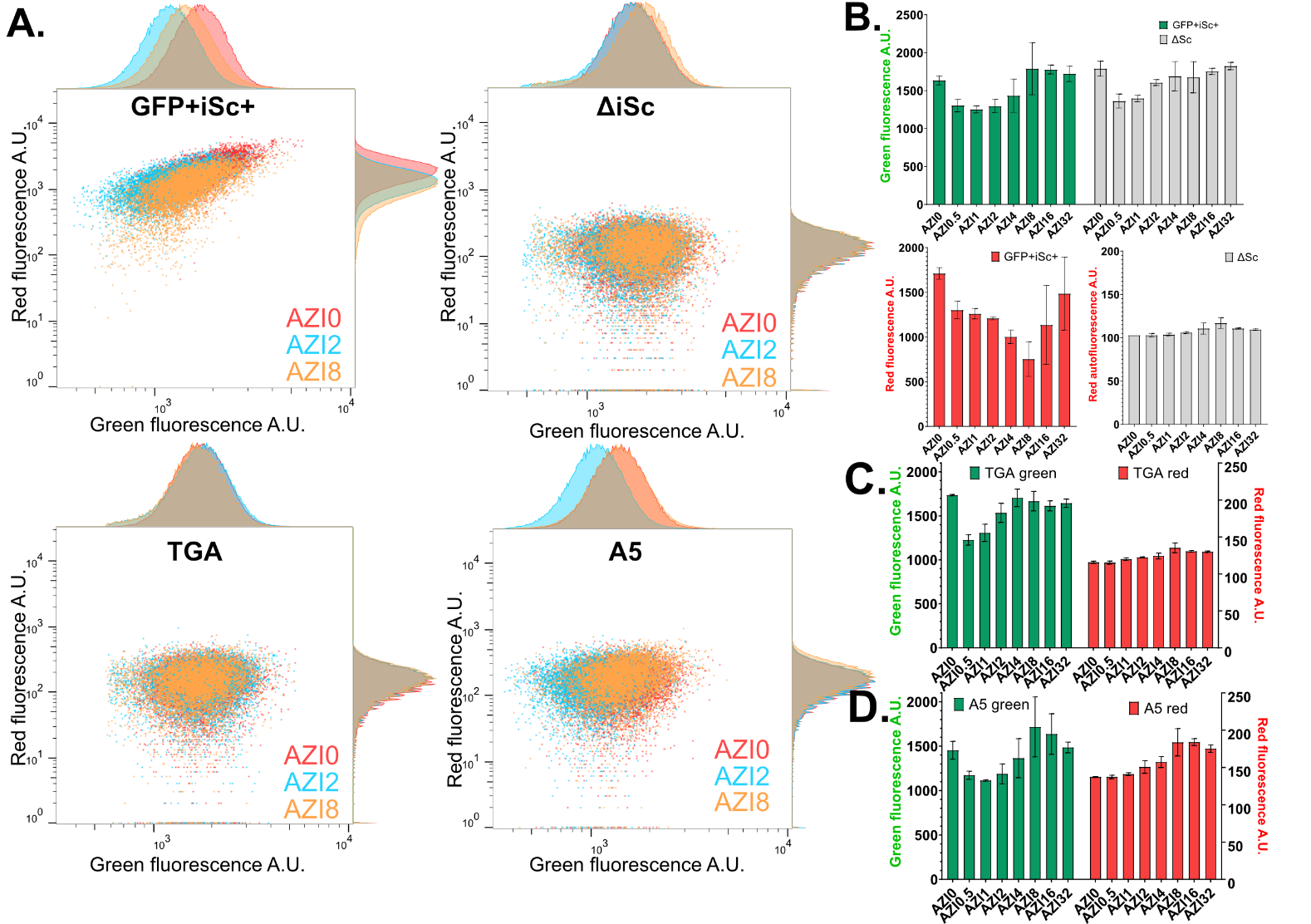


Figure S17. Fluorescence change of gated CFT073 single cells after 4h of treatment with azithromycin (AZI) in MHB. A. Representative green and red fluorescence dotplots show the effect of AZI on fluorescence of controls (GFP+iSc+ and ΔiSc), TGA nonsense and A5 frameshift reporter. 8 µg/ml of AZI (AZI8) GFP+iSc+ sample contains about 8.5% of cells negative for red fluorescence. B. Geometric means of red or green fluorescence of gated control samples in the presence of AZI. Geometric means of red or green fluorescence of gated TGA (C) and A5 (D) reporter samples in the presence of AZI. Red autofluorescence has not been subtracted. N=3


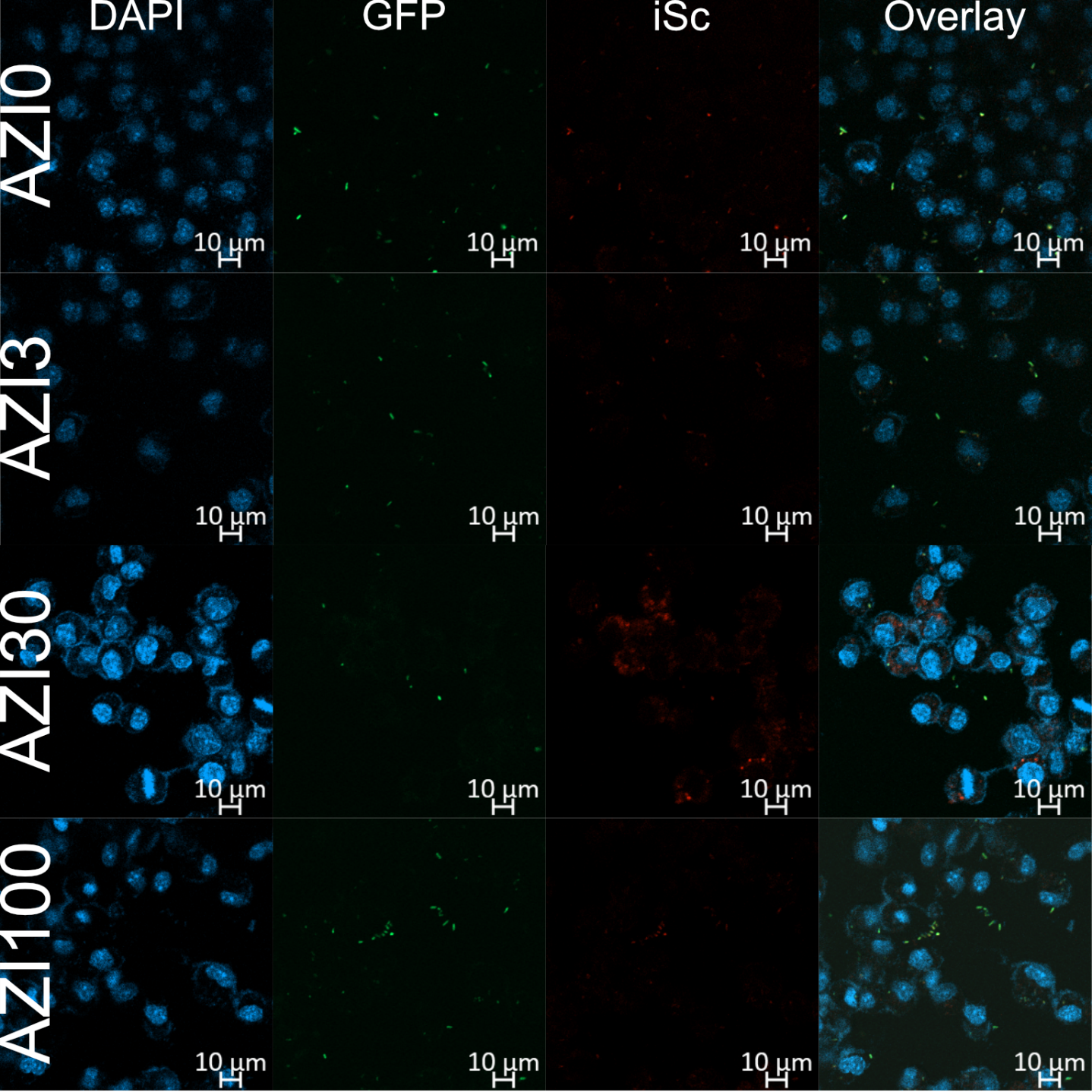


Figure S18. Microscopy images of macrophages infected with uropathogenic CFT073 with frameshift plasmid T7 in the presence of increasing concentrations of azithromycin (AZI). Number indicates AZI concentration in µg/ml. We can detect both GFP and iSc during infection. We see increased red signal inside macrophages at AZI30, however in most cases the signal does not overlap with green signal, which might indicate either very high AZI levels inside macrophages, leading to high mistranslation and/or leakage of bacterial cell contents, such as GFP.

Table S1. List of plasmids used in this paper. In reporter sequences mutation sites are marked in bold red. Stop codons are underlined. All plasmids used carry ampicillin resistance as a selection marker. Plasmids will be made available through Addgene.

| **Plasmid name** | **Abbr.** | **Description (mScarlet-I reporter primary DNA sequence when applicable)** |
| --- | --- | --- |
| *Reporter control plasmids* | | |
| pSC101-AmpR EV | EV | pSC101 empty vector, negative control, autofluorescence |
| pSC101-GFPmut2 (ΔmScarlet) | ΔiSc | No mScarlet-I gene; red negative control, constitutive GFP, mScarlet negative, red autofluorescence |
| pSC101-GFPmut2-mScarlet-I | GFP+iSc+ | constitutive GFP & mScarlet-I, positive control; ATGGTGAGCAAGGGC GAGGCA…225aa…TGA |
| pSC101-GFPmut2-mScarlet-I 6Trp7Ser | 6Trp | constitutive GFP & mScarlet-I with Trp and Ser codons in the mistranslation mutation site, positive control ATGGTGAGCAAGGGC**TGGAGT**GAGGCA…225aa…TGA |
| *Nonsense reporters* | | |
| pSC101-GFPmut2-mScarlet-I TAA | TAA | stop-codon readthrough iSc, constitutive GFP ATGGTGAGCAAGGGC**TAAAGT**GAGGCAGTG |
| pSC101-GFPmut2-mScarlet-I TAG | TAG | stop-codon readthrough iSc, constitutive GFP ATGGTGAGCAAGGGC**TAGAGT**GAGGCAGTG |
| pSC101-GFPmut2-mScarlet-I TGA | TGA | stop-codon readthrough iSc, constitutive GFP ATGGTGAGCAAGGGC**TGAAGT**GAGGCAGTG |
| pSC101-GFPmut2-mScarlet-I TGATC | TGATC | stop-codon readthrough iSc, constitutive GFP  ATGGTGAGCAAGGGC**TGATCT**GAGGCAGTG |
| *Frameshift reporters* | | |
| pSC101-GFPmut2-mScarlet-I A5 | A5 | +1 frameshift iSc, constitutive GFP ATGGTGAGCAAGGGC**AAAAA**GAGGCAGTGA |
| pSC101-GFPmut2-mScarlet-I A6T | A6T | -1 frameshift iSc, constitutive GFP ATGGTGAGCAAGGGC**AAAAAAT**GAGGCAGT |
| pSC101-GFPmut2-mScarlet-I C3T | C3T | -1 frameshift iSc, constitutive GFP ATGGTGAGCAAGGGC**CCCT**GAGGCAGTGAT |
| pSC101-GFPmut2-mScarlet-I T7 | T7 | -1 frameshift iSc, constitutive GFP ATGGTGAGCAAGGGC**TTTTTTT**GAGGCAGT |

Table S2. List of E. coli strains used in this paper.

| **Name** | **Comments** | **Source or reference** |
| --- | --- | --- |
| *Laboratory strains* | | |
| DH5α | Standard laboratory strain |  |
| MG1655 | Standard laboratory strain | Hayashi et al., 2006 |
| BL21 DE3 | Standard laboratory strain for protein production | Stratagene, USA |
| MG1655 ΔL31AB::Km | ribosomal ambiguity mutant strain; with KanR cassette | Lilleorg et al., 2017 |
| MG1655 ΔL31 | MG1655 ΔL31AB::Km, where KanR cassette was removed with pCP20 according to protocol (Datsenko & Wanner, 2000) | This study |
| MC361 | F^−^ *ara* Δ(*gpt-lac*)*5 thi prfB* (from *E. coli* B), WT reference for the following S4, S5 and S12 mutant strains | Agarwal et al., 2015; Agarwal et al., 2011 |
| S4 *ram* | MC361 *rpsD* E201*, ribosomal ambiguity mutant |  |
| S4 *res* | MC361 *rpsD* G86C, error-restrictive mutant |  |
| S5 *ram* | MC361 *rpsE* A109V, ribosomal ambiguity mutant |  |
| S5 *res* | MC361 *rpsE* I104L, error-restrictive mutant |  |
| S12 *res* | MC361 *rpsL* K42T, error-restrictive mutant |  |
| *Clinical isolates* | | |
| CFT073 | Uropathogen; isolated before 1989 | Mobley et al., 1990 |
| Nissle | Also known as Nissle 1917 and EcN; Probiotic; isolated in 1917 | Mutaflor®, Germany |
| DSM1103 | Clinical isolate; EUCAST* quality control strain for susceptibility testing of bacteria to antibiotics; isolated in 1946 | DSMZ, Germany |

*EUCAST - European Committee on Antimicrobial Susceptibility Testing

Table S3. Conditional MICs of antibiotics used in this paper. MIC was determined according to experimental procedure, i.e high inoculum, shaking during incubation, in relevant medium.

| **Antibiotic** | ***E. coli* strain** | **Medium** | **Inhibitory concentration µg/ml** |
| --- | --- | --- | --- |
| Amikacin | MG1655+ pSC101-GFPmut2-mScarlet-I | M9 with 0.2% glucose | 4 |
| Streptomycin |  |  | 8 |
| Kanamycin |  |  | 8 |
| Tobramycin |  |  | 0.5 |
| Apramycin |  |  | 8 |
| Azithromycin* | CFT073 | MHB (cation-adjusted) | 8 |
|  |  | MHB (cation-adjusted) with 25 mM bicarbonate | 0.5 |

*Azithromycin’s conditional MIC determination was not possible due to high inoculum. MIC according to standard procedure [1] is shown.

Table S4. Mistranslation levels of MC361-derived ram and res mutant strains after 24 h of incubation in MHB medium based on platereader data analysis. Means and standard deviations (3 biological replicates) of calculated mistranslation are shown in percentages. P value of difference to WT strain. Statistically significant P values (α<0.05) have been marked in bold.

| **NONSENSE** | | | | | | |
| --- | --- | --- | --- | --- | --- | --- |
|  | **TGA** | | **TAA** | | **TAG** | |
|  | Mean ± SD | P value | Mean ± SD | P value | Mean ± SD | P value |
| WT | 2.9 ± 0.05 |  | 1.9 ± 0.1 |  | 2.5 ± 0.08 |  |
| S4 res | 2.4 ± 0.07 | **<0.001** | 1.9 ± 0.22 | 0.758 | 2.6 ± 0.22 | 0.511 |
| S5 res | 2.3 ± 0.08 | **<0.001** | 1.8 ± 0.16 | 0.253 | 2.5 ± 0.12 | 0.392 |
| S12 res | 1.9 ± 0.14 | **0.004** | 1.7 ± 0.07 | 0.057 | 2.8 ± 0.12 | 0.079 |
| S4 ram | 9.8 ± 0.32 | **<0.001** | 2.1 ± 0.27 | 0.419 | 3.4 ± 0.19 | **0.009** |
| S5 ram | 4.6 ± 0.08 | **<0.001** | 1.9 ± 0.01 | 0.88 | 2.6 ± 0.07 | 0.21 |
| **FRAMESHIFT** | | | | | | |
|  | **T7** | | **A5** | | **C3T** | |
|  | Mean ± SD | P value | Mean ± SD | P value | Mean ± SD | P value |
| WT | 6.7 ± 0.05 |  | 4.8 ± 0.12 |  | 3 ± 0.01 |  |
| S4 res | 7.4 ± 0.28 | **0.039** | 4.9 ± 0.11 | 0.308 | 2.7 ± 0.07 | **0.011** |
| S5 res | 7.1 ± 0.1 | **0.006** | 4.7 ± 0.2 | 0.5 | 2.7 ± 0.09 | **0.021** |
| S12 res | 7.7 ± 0.58 | 0.09 | 4.4 ± 0.21 | 0.056 | 2.3 ± 0.05 | **0.001** |
| S4 ram | 6.3 ± 0.13 | **0.035** | 5.8 ± 0.33 | **0.022** | 5.5 ± 0.51 | **0.014** |
| S5 ram | 6.7 ± 0.47 | 0.983 | 5.4 ± 0.27 | 0.051 | 3.9 ± 0.16 | **0.01** |

Table S5. Summary of mistranslation levels of different E. coli strains in different media after 18 h of incubation based on flow cytometry data analysis. Means and standard deviations of calculated mistranslation are shown in percentages. P value 1 of difference to MG1655 strain. P value 2 of difference to relevant strain in MHB medium. Statistically significant P values (α<0.05) have been marked in bold.

|  |  | **MHB** | | **Human urine** | | **M9** | |
| --- | --- | --- | --- | --- | --- | --- | --- |
| **Strain** | **Reporter** | **Mean ± SD** | **P value 1** | **Mean ± SD** | **P value** | **Mean ± SD** | **P value 2** |
| MG1655 | A5 | 2.4 ± 0.15 |  | 4.3 ± 0.65 | **0.034** | 3 ± 0.16 | **0.006** |
|  | A6T | 0.7 ± 0.06 |  | 0.8 ± 0.18 | 0.496 | 0.4 ± 0.02 | **0.008** |
|  | C3T | 1.5 ± 0.14 |  | 3.1 ± 0.45 | **0.027** | 2.2 ± 0.09 | **0.003** |
|  | T7 | 3.4 ± 0.13 |  | 6 ± 2.6 | 0.225 | 3.1 ± 0.17 | 0.068 |
|  | TAA | 0.8 ± 0.09 |  | 0.9 ± 0.27 | 0.630 | 0.9 ± 0 | 0.293 |
|  | TAG | 1 ± 0.05 |  | 1.1 ± 0.23 | 0.478 | 1.2 ± 0.08 | **0.020** |
|  | TGA | 1.3 ± 0.11 |  | 1.7 ± 0.11 | **0.008** | 1.4 ± 0.06 | 0.437 |
|  | TGATC | 1 ± 0.09 |  | 0.8 ± 0.27 | 0.370 | 1.2 ± 0.08 | 0.081 |
| BL21 DE3 | A5 | 3.6 ± 0.09 | **<0.001** |  |  |  |  |
|  | A6T | 0.6 ± 0.14 | 0.306 |  |  |  |  |
|  | C3T | 1.4 ± 0.05 | 0.622 |  |  |  |  |
|  | T7 | 3.1 ± 0.2 | 0.076 |  |  |  |  |
|  | TAA | 1 ± 0.08 | 0.084 |  |  |  |  |
|  | TAG | 1.9 ± 0.13 | **0.004** |  |  |  |  |
|  | TGA | 1.6 ± 0.1 | **0.04** |  |  |  |  |
|  | TGATC | 1.4 ± 0.27 | 0.118 |  |  |  |  |
| DH5α | A5 | 3.7 ± 0.29 | **0.006** | 7.1 ± 1.56 | 0.062 |  |  |
|  | A6T | 0.8 ± 0.12 | 0.643 | 1.4 ± 0.02 | **0.009** |  |  |
|  | C3T | 2.1 ± 0.14 | **0.006** | 4 ± 0.89 | 0.066 |  |  |
|  | T7 | 3.9 ± 0.26 | 0.089 | 10.6 ± 4.07 | 0.105 |  |  |
|  | TAA | 1.1 ± 0.05 | **0.023** | 1.2 ± 0.17 | 0.478 |  |  |
|  | TAG | 1.2 ± 0.13 | 0.164 | 1.7 ± 0.36 | 0.156 |  |  |
|  | TGA | 1.9 ± 0.18 | **0.018** | 2.3 ± 0.33 | 0.138 |  |  |
|  | TGATC | 1.3 ± 0.02 | **0.024** | 1.3 ± 0.01 | N/A |  |  |

|  |  | **MHB** |  | **Human urine** | | **M9** | |
| --- | --- | --- | --- | --- | --- | --- | --- |
| **Strain** | **Reporter** | **Mean ± SD** | **P value** | **Mean ± SD** | **P value** | **Mean ± SD** | **P value** |
| CFT073 | A5 | 2.1 ± 0.18 | 0.191 | 3.4 ± 0.35 | **0.005** |  |  |
|  | A6T | 0.3 ± 0.02 | **0.005** | 0.4 ± 0.02 | N/A |  |  |
|  | C3T | 1.1 ± 0.04 | **0.036** | 1.3 ± 0.13 | 0.074 |  |  |
|  | T7 | 1.8 ± 0.08 | **<0.001** | 7 ± 2.47 | 0.069 |  |  |
|  | TAA | 0.6 ± 0.04 | **0.022** | 0.6 ± 0.06 | >0.999 |  |  |
|  | TAG | 1.3 ± 0.03 | **0.003** | 1.4 ± 0.12 | 0.225 |  |  |
|  | TGA | 1 ± 0.06 | **0.011** | 1 ± 0.08 | >0.999 |  |  |
|  | TGATC | 0.9 ± 0.08 | 0.209 | 0.7 ± 0.04 | 0.074 |  |  |
| DSM1103 | A5 | 2 ± 0.01 | 0.065 | 3.9 ± 0.13 | <0.001 |  |  |
|  | A6T | 0.3 ± 0.05 | **<0.001** | 0.4 ± 0.11 | 0.288 |  |  |
|  | C3T | 0.9 ± 0.01 | **0.015** | 1.2 ± 0.12 | **0.035** |  |  |
|  | T7 | 1.8 ± 0.04 | **<0.001** | 6.4 ± 2.16 | 0.069 |  |  |
|  | TAA | 0.4 ± 0.02 | **0.014** | 0.6 ± 0.03 | N/A |  |  |
|  | TAG | 1 ± 0.02 | 0.238 | 1.5 ± 0.16 | **0.049** |  |  |
|  | TGA | 0.8 ± 0.01 | **0.012** | 1 ± 0.1 | 0.074 |  |  |
|  | TGATC | 0.6 ± 0.03 | **0.015** | 0.7 ± 0.05 | N/A |  |  |
| Nissle | A5 | 2.4 ± 0.05 | 0.924 | 4.5 ± 0.77 | **0.045** |  |  |
|  | A6T | 0.4 ± 0.02 | **0.005** | 0.4 ± 0.05 | N/A |  |  |
|  | C3T | 1.1 ± 0.03 | **0.037** | 1.6 ± 0.35 | 0.163 |  |  |
|  | T7 | 1.9 ± 0.02 | **0.002** | 6.7 ± 2.56 | 0.085 |  |  |
|  | TAA | 0.6 ± 0.04 | **0.02** | 0.9 ± 0.17 | 0.122 |  |  |
|  | TAG | 1.2 ± 0 | **0.023** | 1.8 ± 0.44 | 0.122 |  |  |
|  | TGA | 0.9 ± 0.04 | **0.013** | 1.3 ± 0.27 | 0.147 |  |  |
|  | TGATC | 0.8 ± 0.07 | 0.058 | 1.1 ± 0.18 | 0.104 |  |  |

Table S6. Summary of mistranslation levels of E. coli MG1655 in M9 minimal medium (with 0.2% glucose) for 4 h in the presence of amikacin based on flow cytometry data analysis. Means and standard deviations of calculated mistranslation are shown in percentages. P values of differences to MHB medium without amikacin. Statistically significant P values (α<0.05) have been marked in bold.

|  |  | **FRAMESHIFT REPORTERS** | | | |
| --- | --- | --- | --- | --- | --- |
| **AMIKACIN**  **µg/ml** |  | **A5** | **A6T** | **C3T** | **T7** |
| 0 | Mean ± SD | 2.7 ± 0.36 | 0.3 ± 0.06 | 1.5 ± 0.23 | 1.4 ± 0.15 |
| 0.5 | Mean ± SD | 2.6 ± 0.27 | 0.2 ± 0.09 | 1.4 ± 0.06 | 1.3 ± 0.09 |
|  | P value | 0.914 | 0.334 | 0.498 | 0.281 |
| 1 | Mean ± SD | 2.9 ± 0.24 | 0.2 ± 0.08 | 1.5 ± 0.09 | 1.4 ± 0.03 |
|  | P value | 0.474 | 0.439 | 0.752 | 0.454 |
| 2 | Mean ± SD | 3.6 ± 0.53 | 0.4 ± 0.03 | 1.9 ± 0.19 | 1.8 ± 0.19 |
|  | P value | 0.063 | 0.067 | 0.094 | 0.081 |
| 4 | Mean ± SD | 3.4 ± 0.39 | 0.4 ± 0.1 | 1.9 ± 0.04 | 1.6 ± 0.15 |
|  | P value | 0.062 | 0.193 | 0.091 | 0.261 |
| 8 | Mean ± SD | 3.7 ± 0.43 | 0.4 ± 0.18 | 1.9 ± 0.19 | 1.5 ± 0.28 |
|  | P value | **0.036** | 0.26 | 0.078 | 0.623 |
|  |  | **NONSENSE REPORTERS** | | | |
| **AMIKACIN**  **µg/ml** |  | **TAA** | **TAG** | **TGA** | **TGATC** |
| 0 | Mean ± SD | 0.6 ± 0.1 | 0.7 ± 0.1 | 1 ± 0.05 | 0.5 ± 0.08 |
| 0.5 | Mean ± SD | 0.4 ± 0.08 | 0.7 ± 0.03 | 1 ± 0.06 | 0.5 ± 0.18 |
|  | P value | 0.135 | 0.411 | 0.198 | 0.872 |
| 1 | Mean ± SD | 0.6 ± 0.12 | 0.7 ± 0.12 | 1.1 ± 0.02 | 0.6 ± 0.03 |
|  | P value | 0.917 | 0.757 | 0.059 | 0.182 |
| 2 | Mean ± SD | 0.8 ± 0.06 | 1 ± 0.12 | 1.4 ± 0.18 | 1.1 ± 0.13 |
|  | P value | **0.024** | **0.034** | 0.05 | **0.005** |
| 4 | Mean ± SD | 0.9 ± 0.07 | 1 ± 0.02 | 1.3 ± 0.1 | 1.2 ± 0.11 |
|  | P value | **0.014** | **0.036** | **0.021** | **0.002** |
| 8 | Mean ± SD | 0.8 ± 0.15 | 1.1 ± 0.2 | 1.4 ± 0.04 | 1.2 ± 0.14 |
|  | P value | 0.069 | 0.085 | **<0.001** | **0.004** |

Table S7. Summary of mistranslation levels of uropathogenic E. coli CFT073 in MHB medium for 4 h in the presence of azithromycin (AZI) based on flow cytometry data analysis. Means and standard deviations of calculated mistranslation are shown in percentages. P values of differences to MHB medium without AZI. Statistically significant P values (α<0.05) have been marked in bold.

|  |  | Frameshift reporters | | | |
| --- | --- | --- | --- | --- | --- |
| AZI µg/ml |  | **A5** | **A6T** | **C3T** | **T7** |
| 0 | Mean ± SD | 2.41 ± 0.13 | 0.31 ± 0.02 | 1.13 ± 0.05 | 1.33 ± 0.07 |
| 0.5 | Mean ± SD | 3.19 ± 0.29 | 0.22 ± 0.05 | 1.26 ± 0.09 | 1.65 ± 0.12 |
|  | P value | **0.028** | 0.074 | 0.113 | **0.025** |
| 1 | Mean ± SD | 3.63 ± 0.21 | 0.22 ± 0.03 | 1.37 ± 0.08 | 1.67 ± 0.06 |
|  | P value | **0.002** | **0.017** | **0.017** | **0.003** |
| 2 | Mean ± SD | 4.39 ± 0.42 | 0.2 ± 0.14 | 1.46 ± 0.27 | 2.02 ± 0.25 |
|  | P value | **0.01** | 0.306 | 0.165 | **0.033** |
| 4 | Mean ± SD | 5.57 ± 1.25 | 0 ± 0.31 | 1.34 ± 0.37 | 1.85 ± 0.32 |
|  | P value | **0.047** | 0.225 | 0.43 | 0.1 |
| 8 | Mean ± SD | 12.16 ± 6.11 | 0.45 ± 1.32 | 2.3 ± 1.21 | 4.14 ± 2.57 |
|  | P value | 0.11 | 0.871 | 0.236 | 0.199 |
| 16 | Mean ± SD | 9.04 ± 4.29 | 0.7 ± 0.06 | 2.39 ± 1.04 | 4.47 ± 3.13 |
|  | P value | 0.116 | **0.004** | 0.17 | 0.224 |
| 32 | Mean ± SD | 5.94 ± 2.15 | 0.76 ± 0.69 | 1.87 ± 0.66 | 2.35 ± 0.68 |
|  | P value | 0.104 | 0.376 | 0.191 | 0.12 |
|  |  | **NONSENSE REPORTERS** | | | |
| AZI µg/ml |  | **TAA** | **TAG** | **TGA** | **TGATC** |
| 0 | Mean ± SD | 0.52 ± 0.05 | 1.24 ± 0.07 | 0.74 ± 0.08 | 0.79 ± 0.05 |
| 0.5 | Mean ± SD | 0.66 ± 0.09 | 1.78 ± 0.14 | 1.09 ± 0.17 | 0.85 ± 0.11 |
|  | P value | 0.096 | **0.01** | 0.052 | 0.457 |
| 1 | Mean ± SD | 1.58 ± 1.46 | 1.99 ± 0.11 | 1.35 ± 0.11 | 0.98 ± 0.11 |
|  | P value | 0.335 | **0.001** | **0.002** | 0.078 |
| 2 | Mean ± SD | 0.71 ± 0.08 | 1.96 ± 0.29 | 1.26 ± 0.13 | 0.98 ± 0.14 |
|  | P value | **0.033** | **0.043** | **0.007** | 0.131 |

Table continues

|  |  | NONSENSE REPORTERS | | | |
| --- | --- | --- | --- | --- | --- |
| AZI µg/ml |  | **TAA** | **TAG** | **TGA** | **TGATC** |
| 4 | Mean ± SD | 0.51 ± 0.25 | 1.51 ± 0.28 | 1.25 ± 0.14 | 0.57 ± 0.18 |
|  | P value | 0.952 | 0.233 | **0.01** | 0.161 |
| 8 | Mean ± SD | 2.02 ± 1.43 | 3.92 ± 1.83 | 3.6 ± 2.14 | 2.41 ± 1.39 |
|  | P value | 0.211 | 0.126 | 0.146 | 0.181 |
| 16 | Mean ± SD | 1.44 ± 0.89 | 3.24 ± 1.77 | 2.55 ± 1.41 | 1.94 ± 1.2 |
|  | P value | 0.215 | 0.189 | 0.156 | 0.239 |
| 32 | Mean ± SD | 0.94 ± 0.25 | 2.1 ± 0.63 | 1.63 ± 0.4 | 1.23 ± 0.32 |
|  | P value | 0.095 | 0.14 | 0.056 | 0.137 |
